# Differential Host Gene Expression Associated with Non-*Lactobacillus*-dominant Vaginal Microbiomes During Pregnancy

**DOI:** 10.21203/rs.3.rs-9268667/v1

**Published:** 2026-05-25

**Authors:** Mallory E. Cunningham, Megan T. Williams, Katherine M. Spaine, Annamaria Marcinkowska, Andrey V. Matveyev, Brian J. Hur, Heather O. Natterer, Rhea B. Chatterjee, Christopher J. Oldfield, Laahirie Edupuganti, Bin Zhu, Kimberly K. Jefferson, Jerome F. Strauss, Amy L. Olex, Myrna G. Serrano, Gregory A. Buck

**Affiliations:** 1 Department of Microbiology and Immunology, Virginia Commonwealth University, Richmond, VA, United States; 2 College of Humanities and Sciences, Virginia Commonwealth University, Richmond, VA, United States; 3 Massey Cancer Center, Virginia Commonwealth University, Richmond, VA, United States; 4 Department of Obstetrics and Gynecology, Virginia Commonwealth University Medical Center, Richmond, VA, United States; 5 Center for Microbiome Engineering and Data Analysis, Virginia Commonwealth University, Richmond, VA, United States of America; 6 Department of Obstetrics and Gynecology, University of Pennsylvania Health System, Philadelphia, PA, United States of America; 7 Wright Center for Clinical and Translational Research, Virginia Commonwealth University, Richmond, VA, United States

**Keywords:** Bacterial vaginosis, metatranscriptomics, pregnancy, RNA-seq, vaginal microbiome, inflammasome, *Gardnerella*, *Lactobacillus*

## Abstract

**Background:**

The vaginal microbiome significantly influences gynecological and obstetric health, yet the interrelationship between host vaginal gene expression and the microbiota during pregnancy is understudied—particularly in racioethnically diverse cohorts. Here, we leveraged metatranscriptomic data from 123 participants from the Multi-Omic Microbiome Study-Pregnancy Initiative (MOMS-PI) cohort to perform a novel integrated analysis of human host gene expression and vaginal microbiota composition during pregnancy. We hypothesized that host gene expression at the vaginal-mucosal interface would exhibit distinct transcriptional profiles when colonized by bacteria commonly present in bacterial vaginosis (BV), termed BV-associated vagitypes, compared to *Lactobacillus*-dominated microbiomes. Such distinct host response would provide evidence linking vaginal inflammation to microbiome composition during pregnancy in a majority Black cohort. By profiling host expression with different BV-associated vagitypes, these host-microbiome signatures could inform clinically actionable biomarkers for microbiome-focused interventions during pregnancy in historically underrepresented populations.

**Results:**

Host transcriptomic profiles differed significantly between BV-associated and *Lactobacillus*-dominated vagitypes, with this association remaining significant when analyses were restricted to Black participants. We identified 13 consistently differentially expressed genes in women with BV-associated vagitypes—vaginal microbiomes comprised of high relative abundance of either *Gardnerella* spp., *Candidatus* Lachnocurva vaginae, or a mixture of multiple anaerobic taxa—compared to women with *Lactobacillus crispatus* vagitypes. These differentially expressed genes are involved in host immune response (*DKK1, H2BC21, ILRUN, S100A9*), oxidative stress response and inflammasome activation (*CSTB*), transcription modulation (*CLK1, PAX9*), vesicle trafficking (*EXPH5*), ubiquitination (*FBXO32*), membrane integrity (*PIEZO1*), and ion transport (*S100A16, SCNN1A, SLCO4A1*).

**Conclusion:**

BV-associated vagitypes are correlated with distinct host immunomodulatory gene expression profiles during pregnancy, independent of self-reported racioethnicity. We demonstrated novel molecular insights into microbiome-host interaction during pregnancy within the context of adverse cervicovaginal health.

## Background

The human microbiome consists of trillions of microorganisms inhabiting diverse body niches involved in various aspects of host physiology and health. Although the vaginal microbiome is typically dominated by one or few bacterial taxa, it exhibits substantial temporal variability across the female life course, being influenced by various factors including diet, hygiene, hormonal status, and genetics [1–3]. Similar to the gut and oral microbiomes, the vaginal microbiome is increasingly recognized for its role in health and disease. Generally, a more homogeneous *Lactobacillus*-dominated microbiome is associated with favorable cervicovaginal health, as the lactobacilli produce lactic acid which maintains an acidic environment (pH ≤ 4.5) that both reduces colonization of the cervicovaginal cavity by pathogens that cause sexually transmitted infections (STI) and promotes mucosal barrier integrity [4, 5]. *Lactobacillus iners* (*L. iners*)-dominated microbiomes represent an intermediate state between optimal and dysbiotic vaginal conditions, as these communities tend to exhibit greater microbial diversity and higher transition rates to non-*Lactobacillus*-dominated states compared to *Lactobacillus crispatus* (*L. crispatus*)-dominated microbiomes [6].

Conversely, bacterial vaginosis is defined as a shift in the vaginal microbiota to colonization by diverse, anaerobic taxa and is usually accompanied with symptoms of increased vaginal discharge, odor, and itching/discomfort [7]. However, many women with bacterial vaginosis are asymptomatic [8]. Symptomatic BV treatment is a global economic burden estimated at $4.8 billion annually [9]. BV is often recurrent, with a rate as high as 58% in some studies [9, 10], complicating patient treatment and prolonging medical expense. The high species diversity and elevated bacterial load observed in bacterial vaginosis are associated with adverse health outcomes including increased risk of STI acquisition [11], preterm birth [12, 13], and pelvic inflammatory disease [14].

Microbiomes dominated by anaerobic, non-*Lactobacillus* bacteria can promote adverse cervicovaginal health through elicitation of host pro-inflammatory responses and disruption of mucosal barrier integrity. BV-associated bacteria produce virulence factors and pathogen-associated molecular patterns (PAMPs) that can activate the host innate immune response. The resulting inflammation can increase infiltration of immune cells at the mucosal surface, enhancing susceptibility to infection with Human Immunodeficiency Virus (HIV) [15, 16]. Women with clinically diagnosed BV exhibit significantly elevated cervicovaginal levels of pro-inflammatory mediators, including, IL-1β, IL-8, IL-6, TNF-α, and human β-defensin, across multiple non-pregnant cohorts of diverse racioethnic backgrounds [11, 17–26]. Even in the absence of clinical BV diagnosis, pregnant women with *non-Lactobacillus*-dominant vaginal microbiomes exhibit elevated pro-inflammatory cytokine levels compared to those with communities dominated by lactobacilli, as demonstrated across racioethnically diverse cohorts [12, 27–30]. Cervicovaginal inflammation has been associated with increased preterm birth risk, with some studies linking this to microbiome composition [27, 28] while others identify cervicovaginal inflammation as an independent risk factor [30].

BV-associated bacteria induce inflammatory cytokine responses in several *in vitro* systems, including immortalized and primary cervical and vaginal epithelial cell lines, immune cell lines, and three-dimensional organoid models [17, 24, 25, 28, 31–35]. Consistent with proteomic and immunoassay data, *in vivo* transcriptomic studies of non-pregnant women have linked *non-Lactobacillus*-dominant cervicovaginal microbiomes with upregulated pro-inflammatory immune response genes in cohorts of varying racioethnicities [19, 36–39]. In contrast, pregnancy transcriptomic studies have focused primarily on peripheral blood or perinatal tissues (umbilical cord, fetal membranes, and placenta) with limited microbiome integration [40, 41]. This represents a significant gap, as ascending cervicovaginal infection is a major contributor to intrauterine infection and preterm birth [42]. Due to the association between *non-Lactobacillus*-dominant cervicovaginal microbiomes and increased neutrophil counts at the cervical-vaginal interface in pregnant women [43], it has been speculated that premature neutrophil recruitment and/or activation via cervicovaginal bacteria may dysregulate normal cervical ripening and contribute to adverse pregnancy outcomes [44]. Understanding how vaginal microbiome composition influences host inflammatory gene expression during pregnancy is therefore critical for identifying microbial drivers of adverse pregnancy outcomes.

Although integrated vaginal transcriptomic and microbiome studies in pregnant women are limited, a few previous investigations link host vaginal transcriptomic signatures with vaginal microbiome composition. Cheng et al. demonstrated that, in a non-pregnant Swedish cohort, vaginal microRNA profiles distinguished between non-*Lactobacillus* -dominant and *Lactobacillus*-dominant microbiomes [38]. Similarly, Wikström et al. integrated vaginal transcriptomics with metagenomic profiling of the microbiota in a Swedish pregnant cohort. Despite identifying genes that were differentially expressed between term and preterm deliveries, they found no significant differences in microbiome diversity between these two groups [45], highlighting the complexity of these relationships. Lastly, in a small study (n=9) of predominantly White pregnant women in the United Kingdom, Zaki et al. identified significant correlations between neutrophil gene expression clusters and cervicovaginal microbiome diversity [46].

While these studies provide important insights into host-microbiome relationships during pregnancy, they share a critical limitation. Despite the strong association between racioethnicity and microbiome composition of both pregnant and non-pregnant women [13, 47–49], integrated vaginal transcriptomic and microbiome studies have been conducted predominantly in White European cohorts, highlighting the need for similar analyses in diverse populations. Here, we present an integrated analysis of host gene expression and vaginal microbiome composition during pregnancy using paired metatranscriptomic and 16S rRNA gene amplicon sequencing data from a cohort of pregnant women with diverse racioethnicity in Richmond, Virginia [13]. In this analysis we identify distinct host transcriptional profiles associated with specific vagitypes. Notably, participants with BV-associated vagitypes dominated by *Candidatus* Lachnocurva vaginae (*Ca*. L. vaginae)*, Gardnerella* spp., and diverse anaerobic taxa (No Type) exhibited differential expression of immunomodulatory genes compared to participants with *Lactobacillus crispatus*-dominated vagitypes.

## Methods

### Participant Enrollment and Health History Data Collection

Data for this study were derived from participants in the MOMS-PI study at Virginia Commonwealth University (VCU) [13]. This study was approved by the VCU Institutional Review Board (IRB protocol number: HM15527), and all procedures were conducted in accordance with ethical standards for human subjects research. The MOMS-PI study longitudinally sampled pregnant women from enrollment through birth for vaginal microbiome and host profiling to analyze the impact of the vaginal microbiome on host health and pregnancy outcome. Written informed consent was obtained from all participants ≥ 18 years of age prior to enrollment. For participants aged 15–17 years, parental permission and participant assent were obtained. Upon enrollment and at each follow-up visit, both clinical and health survey data were collected. Participants self-reported their racioethnic background in response to the question: “What is your ethnic or racial background (Check all that apply)” within the health history questionnaire.

Samples from the larger MOMS-PI cohort were selected for this study based on the following criteria: (1) one sample per participant for each type of analysis (transcriptomic or cytokine), (2) the sample taken at the earliest gestational age available for each participant, and (3) exclusion of women who self-reported antibiotic use or reported the use of medication indicating a significant health condition including diabetes, organ transplantation, or viral infection via health survey at the time of sampling.

### Sample Collection, Nucleic Acid Extraction, and Ribosomal RNA Depletion

Maternal sampling and RNA and DNA extraction procedures and protocols for generation of 16S rRNA gene amplicon sequencing profiles are described in Fettweis et al., 2019. In brief, mid vaginal swabs from ~5 cm from the vaginal opening were collected by either a clinician or the participant using CultureSwab EZ^™^ swabs. The swabs were agitated in either RNAlater^®^ (Qiagen) for samples allocated for RNA extraction or MoBio PowerSoil DNA^™^ isolation buffer for samples allocated for DNA extraction, and the resulting eluates were stored at −80°C within an hour of collection. The eluate was subjected to RNA extraction with the MoBio PowerMicrobiome RNA Isolation Kit as described by the manufacturer. Ribosomal RNA was removed from the extracted total RNA with the Epicentre/Illumina Ribo-Zero Magnetic Epidemiology Kit as described by the manufacturer and stored at −80°C. DNA was extracted from mid vaginal swab eluate with the MoBio PowerSoil DNA isolation kit as described by the manufacturer.

### 16S rRNA Gene Amplicon Sequencing and Taxonomic Profiling

Extracted vaginal swab DNA was amplified with primers targeting the bacterial 16S V1-V3 rRNA gene region as previously described [13] and sequenced with the Illumina MiSeq using 2×300 paired-end protocols. Raw read processing was performed and the 16S rRNA gene amplicon sequences were taxonomically classified using the STIRRUPS database as previously described [50]. Species relative abundance calculations were performed with in-house scripts https://github.com/MalloryCunninghamVCU/MOMS-PI_WMTS_2025.git. Vagitype designation was assigned to each sample based on the dominant taxon (the taxon with the highest relative abundance > 30%). The No Type vagitypes had no taxon reach a percent relative abundance ≥ 30%. Samples with ≥ 30% relative abundance of the *Gardnerella* genus were deemed *Gardnerella* spp., as V1-V3 16S rRNA gene amplicon sequences cannot differentiate *Gardnerella* species [51–53].

The average 16S rRNA gene amplicon sequencing read count per sample was 81,997 (SD ± 83,771), with a median of 64,869 reads (IQR = 50,110). Library size normalization for alpha diversity calculations was performed with the GUniFrac (v1.8) R package [54] to a fixed read depth of 1000 reads for 100 rounds of repeated rarefaction, similar to the method described in [55]. A phyloseq (v1.52.0) object was created for each round of rarefaction and had a calculation of per sample Shannon diversity, Observed taxonomic unit (OTU) evenness, and number of OTUs across all iterations [56]. The difference in the Shannon diversity index between two factors (e.g. participants with above average *IL-1β* expression vs. those with below average *IL-1β* expression) was assessed using a Mann-Whitney U test, with the Shannon index used as the pre-specified primary metric. The Shannon index, OTU evenness, and number of OTUs Mann-Whitney U test p-values were also corrected together as a group with the Benjamini-Hochberg (BH) p-value adjustment with the stats (v4.5) R package [57]. Beta diversity calculation of Bray-Curtis distances between the two factors was performed with the vegan (v2.7.1) R package [58] with a permutational analysis of variance (PERMANOVA) of the distances with the vegan adonis2 test. To avoid stochastic loss of reads that would alter the apparent dominant taxon in each sample, the stacked bar plots represented in the results show per-sample relative abundances (OTU read count/sample total) computed from the non-rarefied OTU table.

### Metatranscriptomic Library Preparation and Sequencing

Metatranscriptomic libraries from the rRNA-depleted RNA were prepped using the KAPA Biosystems KAPA RNA HyperPrep kit and sequenced on the Illumina HiSeq4000 sequencer using 2 × 150 base paired-end protocols as previously described [13].

### Raw Read Processing and Classification

Raw metatranscriptomic reads were processed for adapter removal and quality trimming with fastp v0.23.4 [59]. The trimmed reads were input into Kraken2 v2.1.3 with the k2_pluspf (2023-10-09) database (https://benlangmead.github.io/aws-indexes/k2.) including additional in-house bacterial genomes for estimation of bacterial and human read proportions [60]. Only samples with at least 20 million paired-end human reads were kept for downstream differential expression analysis. Samples were analyzed for post-processing quality with MultiQC v1.17.

### Human Papillomavirus (HPV) PCR Typing

In brief, DNA extracted from the vaginal swabs was combined with primers for the human papillomavirus major capsid protein L1 gene in a two-step PCR as described in Bridy et al. 2025 [61]. The PCR differentiates the following HPV types: 11, 16, 18, 26, 30, 31, 32, 33, 34, 35, 38, 39, 40, 42, 44, 45, 51, 52, 53, 54, 56, 58, 59, 61, 62, 66, 67, 68, 70, 71, 72, 73, 74, 81, 82, 83, 84, 85, 86, 87, 89, 90, 102, 107, 114, 120, and 124. Post two-step HPV L1 gene amplicons were cleaned with Agencourt AMPure XP (Beckman Coulter Genomics) following the manufacturer’s protocol and pooled equimolarly for sequencing with an Illumina MiSeq platform using the 2 × 300 paired-end protocol. The trimmed and quality filtered reads were mapped to a custom L1 gene database. Samples were considered HPV positive if two criteria were met: (1) greater than ten percent of the total reads for the sample were mapped to any HPV type, and (2) the sample had greater than ten total viral reads. Given the reported median 13.29-month duration of HPV infection [62], a participant found to be HPV positive with the aforementioned criteria at any sampling during their pregnancy was thus considered HPV positive. Patients were asked to self-report their prior history of HPV vaccination in the intake questionnaire with the following statement: “Have you received the HPV Vaccination (Gardasil^®^/Cervarix^®^)?”

### Host Read Alignment and Transcript Quantification

Transcript-level quantification was performed using Salmon v1.10.1 in selective alignment mode (--validateMappings) with sequence and GC bias correction (--seqBias), (--gcBias) and variational Bayesian EM optimization of abundance estimates (--useVBOpt). A decoy-aware Salmon index was constructed using the Ensembl GRCh38 release 102 primary assembly genome and the corresponding Ensembl GRCh38 release 102 cDNA transcriptome. Salmon transcript-level estimates were imported into R and summarized to gene-level count matrices using tximport v1.34 with a transcript-to-gene mapping derived from the corresponding Ensembl GRCh38 release 102 GTF annotation file. The resulting gene-level counts and average transcript lengths were used to construct DESeq2 matrices for differential expression analysis via the DESeqDataSetFromTximport function.

### Differential Gene Expression Analysis with DESeq2

Prior to running DESeq2 [63], we prefiltered genes by retaining those with a raw count of ≥ 10 in at least 2 samples across all 123 samples. Gene name mapping of the Entrez Gene identifiers to HUGO Gene Nomenclature Committee (HGNC) symbols for gene count aggregation was performed with the org.Hs.eg.db R package [64].

The DESeq2 analysis design formula used was: ~Med_final + systolic_scaled + age_scaled + ga_atSampling_weeks_scaled + self-reported racioethnicity + IncomeModified + HPV infection status + vagitype. Systolic blood pressure, gestational age at the time of sampling, and participant age were scaled with the base R scale function [57]. For the “Med_final” classification of participants, acceptable medications taken at the time of sampling included prenatal vitamins, antiemetics, over-the-counter analgesics, progesterone, antacids, iron supplements, antihistamines, Butalbital/Acetaminophen/Caffeine (Fioricet^™^), and low molecular weight heparin (enoxaparin). Women who self-reportedly took no medication at the time of sampling served as the reference level for the ‘Med final’ variable, women with an annual income less than $15,000 served as the reference level for the ‘IncomeModified’ variable, Black women served as the reference level for the ‘Self-reported racioethnicity’ variable, and women with a *L. crispatus* vagitype served as the reference level for the ‘vagitype’ variable. The contribution of each covariate to the gene expression variance between samples via DESeq2 was analyzed with the variancePartition R package [65]. As only one sample was analyzed per person, all covariates were modeled as fixed effects with variancePartition. Correlation between the DESeq2 design covariates was assessed with the variancePartition Canonical Correlation Analysis function ‘canCorPairs’.

Differential expression was assessed using DESeq2 with significance defined as genes with both a Benjamini Hochberg adjusted p-value (FDR) < 0.05 and a baseMean ≥ 10. We did not apply a strict absolute log_2_ fold change threshold as a primary filter because this study was exploratory and included comparisons among vagitype groups of unequal size while adjusting for multiple covariates. Low-information features were instead limited by the baseMean threshold to reduce genes with low average expression.

### Host Inflammatory Gene Expression by Quantitative Reverse Transcription Polymerase Chain Reaction (qRT-PCR)

RNA from vaginal swab samples was reverse transcribed into cDNA with the SuperScript^™^ IV VILO^™^ cDNA Synthesis Kit using random primers (ThermoFisher Scientific). The cDNA was subjected to qRT-PCR using predesigned PrimeTime^™^ Gene Expression Assay probes (Integrated DNA Technologies (IDT)) targeting *IL-1β, CXCL8, BCL2A1* and *NCF2* with Applied Biosystems^™^ Human *ACTB* (Beta Actin, Cat. 4310881E) as the endogenous control. Per sample target gene ΔCt (cycle threshold difference) was calculated by normalizing target gene average Ct by subtraction of the average duplicate *ACTB* expression ΔCt. Fold change between samples was calculated by subtracting the average target ΔCt from the *L. crispatus* vagitype samples from the target ΔCt in the *Ca*. L. vaginae, *Gardnerella* spp., No Type, *L. iners*, and *L. gasseri* vagitypes to recapitulate a fold change value similar to the DESeq2 gene expression calculations. Normality of the distribution of log_2_ fold changes of each vagitype groups’ target gene was assessed via the Shapiro-Wilk test. The Welch analysis of variance (ANOVA) test, which is robust to both different sample sizes and the violation of the homogeneity of variance, was performed to assess the difference in target gene expression across vagitypes with Games-Howell post hoc adjustment for pairwise comparison. qRT-PCR was performed on a subset of the MOMS-PI samples (n = 94) for which sufficient RNA remained after metatranscriptomic library preparation.

### Gene Pathway Analysis with STRINGdb

Overlap between the significantly upregulated and downregulated genes between the *Gardnerella* spp., *Ca*. L. vaginae, and No Type vagitypes with the *L. crispatus* baseline were visualized with Venny in R [66]. Gene pathway enrichment analysis was performed with STRINGdb [67].

### Vaginal Swab Soluble Cytokine Quantification

Vaginal swabs were processed for cytokine profiling as previously described [13]. In brief, vaginal swab samples frozen in 500 ul of 10 mM Tris, pH 7.0, 1 mM EDTA were thawed on ice, centrifuged at 10,000 × g for 10 minutes at 4°C and diluted in 100 mM Tris buffer, pH 7.5. Samples and 50 ul of cytokine standard were added to a black 96-well plate and read with the Bio-Plex MAGPIX Multiplex Reader with default settings and v.6.0 software. Picograms of IL-1β were normalized to the total protein concentration of each plate measured in milligrams. The pg IL-1β/mg total protein was transformed by taking the natural log of the IL-1β measurement and the normality assessed with the Shapiro-Wilk test.

## Results

### Characterization of Participant Vaginal Microbiomes

**Figure 1.**
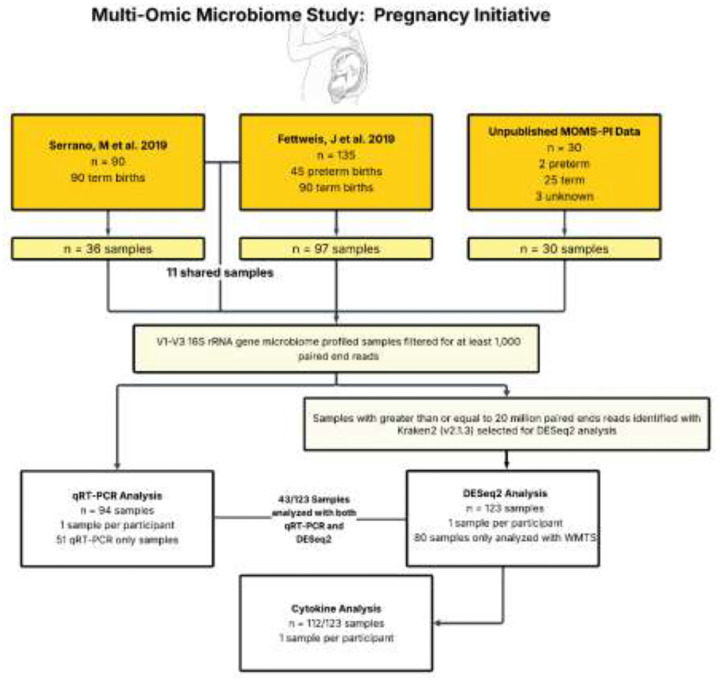
Overview of Study Data Acquisition and Processing Samples analyzed in this study were drawn from the MOMS-PI raw data generated in the Serrano, et al. 2019 and Fettweis, et al. 2019 studies, as well as data from previously unpublished samples. WMTS = Whole metatranscriptomic sequencing.

A total of 123 paired 16S rRNA gene amplicon sequence and host transcriptomic profiles from participants of the MOMS-PI cohort were analyzed in this study, including both previously characterized samples and previously unpublished samples (Figure 1) [13, 48].

The participants included in the DESeq2 analysis were predominantly Black women (83/123, 67.5%), women who later delivered at term (90/123, 73.2%), and women who were sampled in their second trimester (98–182 days of gestational age at sampling) (Table 1). White participants were significantly older than their Black counterparts utilizing both a Kruskal-Wallis Rank Sum test and a subsequent pairwise comparisons with a one-sided Wilcoxon rank sum test (Bonferroni adjusted p-value ≤ 0.05).

**Figure 2.**
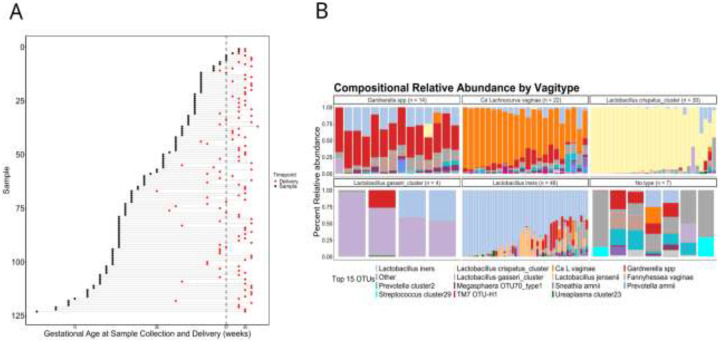
Participant Vagitypes and Gestational Age at the Time of Sampling (**A**) Sample distance dot plot of participant gestational age at the time of sample collection and delivery. Each black dot represents a collection timepoint of the mid-vaginal wall swabs used for V1-V3 16S rRNA gene amplicon sequencing and metatranscriptomic sequencing for this cohort (n =123). The horizontal lines connect the gestational age of the participant at the time of sampling to their gestational age at the time of delivery in weeks. The vertical line at 37 weeks demarcates preterm delivery (delivery <37 gestational weeks) from term delivery. (**B**) Vaginal microbiome V1-V3 16S rRNA gene amplicon sequencing profile at the time of sample collection. The sample relative abundance proportions in the stacked bar plot are derived from the non-rarefied proportions.

The DESeq2 analysis formula accounted for relevant participant covariates including demographic information (self-reported racioethnicity, age, annual income), clinical variables (medication use, systolic blood pressure, infection with HPV), and pregnancy-related information (gestational age) at the time the samples were collected. Canonical correlation analysis between individual covariates and the percentage of the variation in gene expression data explained by each covariate within the DESeq2 model are shown in **Supplementary Figure 1**. There was a weak correlation between participant vagitype and age (absolute canonical correlation coefficient (CCA) |ρ| = 0.33) and between vagitype and racioethnicity (CCA |ρ| = 0.31). Additionally, there was a weak correlation between income and age (CCA |ρ| = 0.36) and income and racioethnicity (CCA |ρ| = 0.35). Only one sample per participant, specifically the sample collected at the earliest timepoint available in each participant’s pregnancy, was analyzed for differential gene expression with DESeq2 (Figure 2A). The average weeks between participant sample collection and participant delivery (ΔGA) was 11.4 weeks (SD = 5.20) for preterm birth participants and ΔGA = 14.1 weeks (SD = 8.01) weeks for term birth participants. Vaginal microbiome composition was determined by 16S rRNA gene amplicon sequencing of the V1-V3 region as previously characterized [13]. The most common vagitypes in order of descending occurrence were, *Lactobacillus iners* (*L. iners*), *L. crispatus*, *Ca*. L. vaginae, *Gardnerella* spp., No Type, and *Lactobacillus gasseri* (*L. gasseri*) (Figure 2B).

### Distinct Host Vaginal Gene Expression Profiles of BV-Associated Vagitypes Compared to *L. crispatus*

Homogeneous vaginal microbiomes with high relative abundance of *Lactobacillus* species, particularly *L. crispatus*, are generally associated with vaginal health due to their exclusion of pathogens. Therefore, participants with the *L. crispatus* vagitype were selected as the reference group for DESeq2 pairwise transcriptomic comparisons. Participants with the *Gardnerella* spp. and *Ca*. L. vaginae vagitypes were comprised of exclusively (100%) and mainly (81.8%) Black participants, respectively (Table 1). Participants with the *L. crispatus* baseline vagitype were split between Black and White women, with a few samples from participants with the Hispanic/Latina or Mixed racioethnicity (Table 1). Gestational age at the time of sampling was not significantly different among the vagitype groups in either the Kruskal-Wallis (p = 0.63) or pairwise Wilcoxon tests with Bonferroni correction (p_adj_ = 1.0).

**Figure 3.**
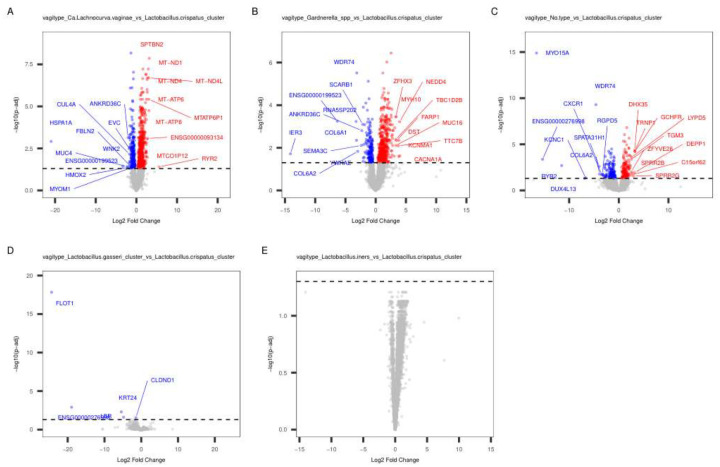
Host Vaginal Gene Expression Between Multiracial Pregnant Women with Contrasting Vaginal Microbiome Compositions (**A**) Volcano plot of n =809 significantly differentially expressed genes between participants with *Ca*. L. vaginae (n = 22) vagitypes compared to participants with the *L. crispatus* vagitype (n = 30). (**B**) Volcano plot of n =656 significantly differentially expressed genes between participants with the *Gardnerella spp*. (n =14) vagitype compared to participants with the *L. crispatus* vagitype. (**C**) Volcano plot of n =315 significantly differentially expressed genes between participants with the No Type (n = 7) vagitype compared to participants with the *L. crispatus* vagitype (**D**). Volcano plot of n = 5 differentially expressed genes between participants with the *L. gasseri* (n = 4) vagitype compared to participants with the *L. crispatus* vagitype. (**E**) Volcano plot of n =0 significant differentially expressed gene between participants with the *L. iners* (n =46) vagitype compared to participants with the *L. crispatus* vagitype. In each volcano plot, the denominator dictating the directionality of the log_2_ fold values is *L. crispatus*. P-values for significantly differentially expressed genes were adjusted with the Benjamini-Hochberg method. Significance = p_adj_ < 0.05, baseMean >10.

The expression profiles of three BV-associated vagitype groups were compared to participants with the *L. crispatus* vagitype (n =30): *Gardnerella* spp. (n =14), *Ca*. L. vaginae (n =22), and No Type (n =7). This analysis also included the analysis of host expression with *Lactobacillus* dominant vagitypes compared to *L. crispatus*: *L. iners* (n =46) and *L. gasseri* (n = 4). Women with the *Ca*. L. vaginae vagitype had the largest number of significantly differentially expressed genes (sDEG) from the *L. crispatus* vagitype (sDEG = 809, Figure 3A, **Supplementary Table S2**), followed by *Gardnerella* spp. (sDEG = 656, Figure 3B, **Supplementary Table S3**), No Type (sDEG = 315, Figure 3C, **Supplementary Table S4**), *L. gasseri* (sDEG = 5, Figure 3D, **Supplementary Table S5**), and lastly *L. iners* (sDEG = 0, Figure 3E). In contrast to other vagitypes, there were zero differentially regulated gene between participants with the *L. iners* vagitype compared to *L. crispatus* (Figure 3E). These findings demonstrate that participants with BV-associated vagitypes have distinct host transcriptional profiles compared to *L. crispatus*-dominated microbiomes.

### Inflammasome and NF-kB Pathway Genes are Differentially Expressed within BV-Associated Vagitypes Compared to *L. crispatus*

Seven notable genes associated with inflammasome and NF-kB pathways were differentially expressed within BV-associated vagitypes compared to *L. crispatus* vagitypes (Table 2), particularly in participants with the *Ca*. L. vaginae vagitype.

### Common Differentially Expressed Genes Among BV-Associated Vagitypes

**Figure 4.**
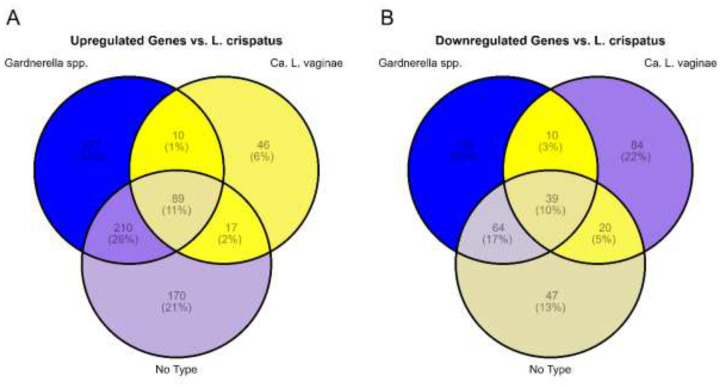
Gene Overlap Between BV-associated Vagitypes Compared to Participants with *L. crispatus* Vagitypes (**A**)Overlapping significantly upregulated genes between *Ca*. L. vaginae, *Gardnerella* spp., and No Type vagitypes versus *L. crispatus* vagitypes. (**B**) Overlapping significantly downregulated genes between *Ca*. L. vaginae, *Gardnerella* spp., and No Type vagitypes versus *L. crispatus* vagitypes. Significance was determined by DEseq2 (p_adj_ < 0.05, baseMean > 10).

To identify common host responses with high relative abundance of different BV-associated microbiome species, we identified gene expression changes common to the three BV-associated vagitypes: *Gardnerella* spp., *Ca*. L. vaginae, and No Type relative to the *L. crispatus* vagitype. We identified 89 genes that were consistently upregulated and 39 genes that were consistently downregulated between the three BV-associated vagitypes relative to the *L. crispatus* vagitype (Figure 4A, **Supplementary Table S6-S7**). Pathway enrichment analysis with StringDB of the 89 upregulated genes identified 11 genes within the *VEGFA-VEGFR2* signaling pathway (WikiPathways WP388 [76]): *MAP2K2, ARF6, P4HB, IDH2, PPM1G, CSRP1, IER5, JUN(AP-1), SHROOM2, GIPC1, EPN1*. This same pathway was previously found to be predictive of preterm birth in a maternal blood multi-omic study including integrated microbiome data [41]. Other notable commonly upregulated genes included: *ILRUN*—which regulates pathogen-stimulated cytokines [77], *IL-22RA1*—which regulates mucosal niche colonization by bacteria [78], *KLF5*—which modulates the lipopolysaccharide proinflammatory response [79], and *TICAM1*—a gene that mediates MYD-88 independent proinflammatory signaling through toll-like receptors (TLR) 3 and 4 [80]. Common downregulated genes included *S100A9*—a gene crucial to epithelial defense during *Candida* infections [81], *STK10*—which participates in lymphocyte migration and microvilli function [82], *PIEZO1*— a mechanical sensor shown to maintain colonic mucosal barriers and microflora [83], *SCEL*—a gene encoding a cornified envelop precursor [84], and *SNX9*—which participates in clathrin-mediated endocytosis and is downregulated in chronic inflammation [85]. Collectively, these findings highlight BV-associated vagitype association with host genes that converge on inflammatory and pathogen response pathways.

### Distinct Host Vaginal Gene Expression Profiles of BV-Associated Vagitypes Compared to *L. crispatus* Independent of Self-Reported Racioethnicity

**Figure 5.**
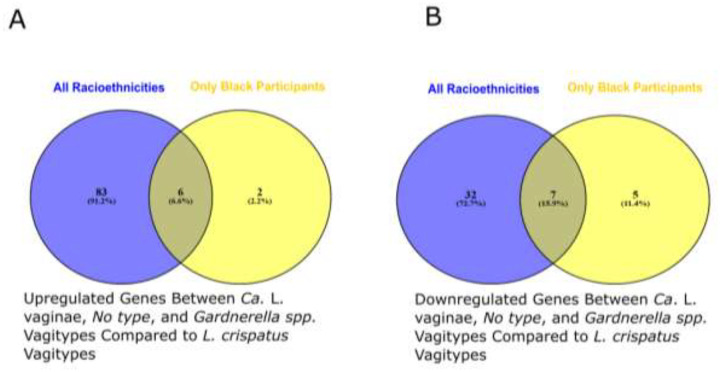
Differentially Expressed Genes Between Bacterial-Vaginosis Associated Vagitypes among the Full and Reduced Cohorts (**A**) Overlapping significantly upregulated genes among pairwise DESeq2 contrasts between BV-associated vagitypes compared to *L. crispatus* in the entire racioethnic cohort (n =123) compared to the reduced cohort consisting of only Black participants (n = 83). (**B**) Overlapping significantly downregulated genes among pairwise DESeq2 contrasts between BV-associated vagitypes compared to *L. crispatus* in the entire multi-racial cohort compared to the reduced cohort consisting of only Black participants. Significance was determined by DEseq2 (Benjamini-Hochberg adjusted p-value ≤ 0.05, baseMean > 10).

To disentangle the effects of self-reported racioethnicity and vagitype on the host transcriptome, participant data were restricted to those from only Black participants (n = 83), thereby removing the confounding between self-reported racioethnicity and the *Gardnerella* spp. and *Ca*. L. vaginae vagitypes. Within this subgroup, and despite the smaller number of samples, the transcriptome profiles from women with *Ca*. L. vaginae, *Gardnerella* spp., and No Type vagitypes remained significantly transcriptionally distinct from those with the *L. crispatus* vagitype (**Supplementary Figure 2**). Genes that continued to be differentially expressed across the three BV-associated vagitypes in this subset were compared to the genes identified in the full racioethnic analysis (Figure 5). The genes differentially expressed in each of the three BV-associated vagitypes, regardless of whether the analysis was limited to just Black women or all racioethnicities, included the following genes: upregulated—EXPH5, H2BC21, ILRUN, PAX9, S100A16, SCNN1A, downregulated—CLK1, SLCO4A1, PIEZO1, DKK1, FBXO32, CSTB, and S100A9. The persistence of these differentially expressed genes in the racioethnicity-restricted analysis underscores the consistency of the immunomodulatory host transcriptomic response in BV-associated vagitypes in this pregnant cohort, independent of racioethnicity.

### Variable Host Gene Pathway Enrichment

**Figure 6.**
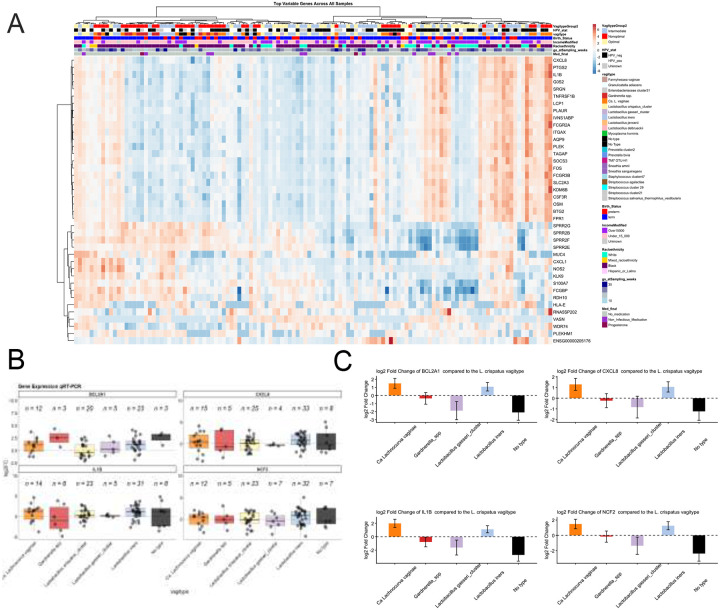
Variably Expressed Immune Genes Among Vagitypes Analyzed Via Quantitative Polymerase Chain Reaction (qPCR) and DESeq2 (**A**) Heatmap of the top 40 most variably expressed genes identified with DESeq2 between n =123 participants using variance-stabilized counts. Values are row-centered to visualize relative expression patterns. (**B**) Boxplots of log_2_ fold change values of four inflammatory response genes across vagitypes (n = 94) compared to *L. crispatus* analyzed via qRT-PCR. (**C**) Bar plots of log_2_ fold change values of four inflammatory response genes across vagitypes compared to *L. crispatus* analyzed via DESeq2 (n = 123).

Differential expression analysis of participant vaginal transcriptomes revealed variability in gene expression between participants (Figure 3). The top forty genes with the largest variance were extracted from the DESeq2 variance stabilizing transformation of the per-gene expression values across all samples, depicted in Figure 6A. Two distinct gene groupings were observed within the heatmap. STRING Functional enrichment analysis of the larger cluster (23 genes: *CXCL8, PTGS2, IL-1β, G0S2, SRGN, TNFRSF1B, LCP1, PLAUR, IVNS1ABP, FCGR2A, ITGAX, AQP9, PLEK, TAGAP, SOCS3, FOS, FCGR3B, SLC2A3, KDM6B, CSF3R, OSM, BTG2, and FPR1*) identified significant enrichment of host acute inflammatory and cytokine response (**GO:0006954** Inflammatory response, **GO:0051716** Cellular response to stimulus, and **GO:0007166** Cell surface receptor signaling pathway. Functional enrichment analysis within the smaller gene pathway cluster (*SPRR2G, SPRR2B, SPRR2F, SPRR2E, MUC4, CXCL1, NOS2, KLK9, S100A7, FCGBP, RDH10, HLA-E, RNA5SP202, VAN, WDR74, PLEKHM1*, and *ENSG00000205176*) displayed enrichment of vaginal epithelial barrier function pathways (**GO:0030216** Keratinocyte differentiation, **GO:0031424** Keratinization, **GO:0009617** Response to bacterium, and **GO:0019730** Antimicrobial humoral response).

### Differential Host Gene Expression Profiles with Quantitative Polymerase Chain Reaction (qRT-PCR) Analysis

To assess the variable gene expression of key immune response/inflammatory markers observed in the DESeq2 data (Fig 6A), we calculated the log_2_ fold expression of four genes with qRT-PCR: *IL-1β, CXCL8, NCF2*, and *BCL2A1* across vagitypes with *ACTB* as the endogenous per-sample control. A full list of participant demographics for the qRT-PCR samples is listed in **Supplementary Table S8**. The log_2_ fold change expression of all four qRT-PCR target genes was generally directionally consistent with the differential expression trends observed in the host transcriptomic profiles (Figure 6B and 6C), supporting the validity of the RNA-seq findings. However, none of the four genes reached statistical significance across vagitypes via Welch ANOVA with the Games-Howell post-hoc adjustment (p_adj_ < 0.05), likely due to reduced statistical power from the smaller qRT-PCR sample size (n = 94) and within-vagitype variability of the log2 fold change values.

### Microbiome Diversity is Associated with IL-1β Gene Expression in *Lactobacillus*-dominant Vagitypes

Given the considerable within-vagitype variability of the log_2_ fold change of the four inflammatory genes observed via qRT-PCR (Figure 6B), we investigated whether microbiome community composition differed between samples with above- and below-average log_2_ fold change *IL-1β* expression in the qRT-PCR, DESeq2, and cytokine datasets similar to methods previously utilized in gut microbiome/cytokine association studies [86–88]. The resulting Mann-Whitney U test between the Shannon diversity of participants with above and below average *IL-1β* expression and the beta diversity measures for each comparison are reported in Table 3 (Table 3).

**Figure 7.**
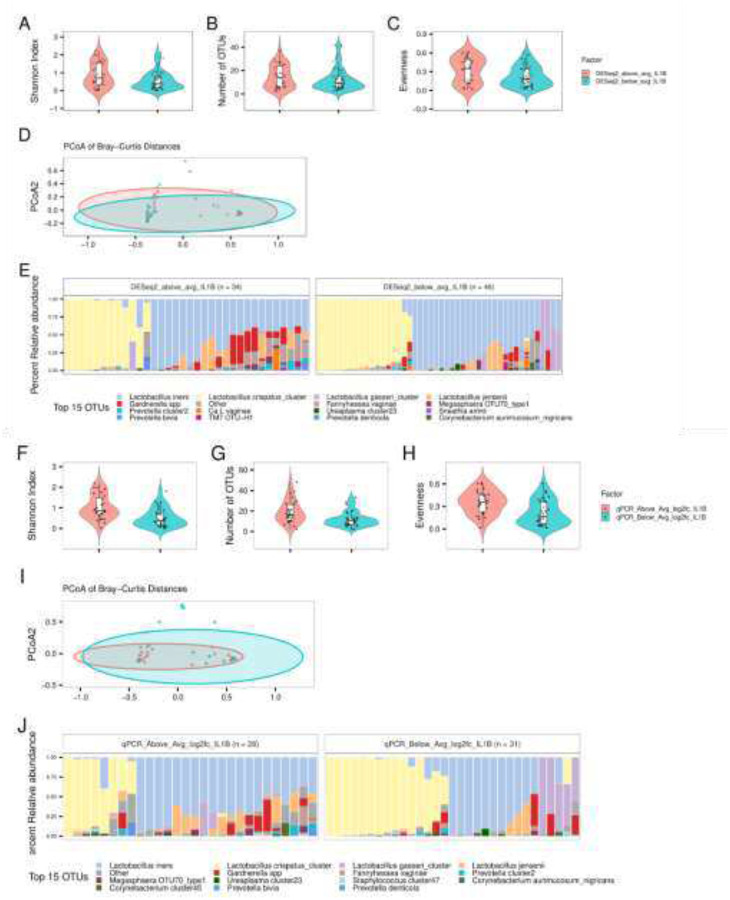
Alpha and Beta Diversity Between Samples with Above and Below Average *IL-1β* Expression (**A**)Violin plot of the Shannon diversity between *Lactobacillus*-dominant vagitypes with above and below average DESeq2 log_2_ fold change in *IL-1β* expression (p = 0.047, BH adj-p = 0.071). (**B**) Violin plot of the observed taxonomic units between *Lactobacillus*-dominant vagitypes with above and below average DESeq2 log_2_ fold change *IL-1β* expression (p = 0.257, BH adj-p = 0.257). (**C**) Violin plot of the evenness between *Lactobacillus*-dominant vagitypes with above and below average DESeq2 log_2_ fold change *IL-1β* expression (p = 0.038, BH adj-p = 0.071). (**D**) Principal component plot of Bray-Curtis distances between *Lactobacillus*-dominant vagitypes with above and below average log_2_ fold change DESeq2 *IL-1β* expression (p = 0.41). (**E**) Stacked bar plot of *Lactobacillus*-dominant vagitype percent relative abundance of taxonomic units within above and below average DESeq2 log_2_ fold change *IL-1β* expression. (**F**) Violin plot of the Shannon diversity between *Lactobacillus*-dominant vagitypes with above and below average qPCR log_2_ fold change *IL-1β* expression (p = 0.0004, BH-adj p = 0.001). (**G**) Violin plot of the observed taxonomic units between above and below average qPCR log_2_ fold change *IL-1β* expression (p = 0.005, BH-adj p = 0.005). (**H**) Violin plot of the evenness between above and below average qPCR log_2_ fold change *IL-1β* expression (p = 0.002, BH-adj p = 0.003). (**I**) Principal component plot of Bray-Curtis distances between above and below average log2 fold change qPCR *IL-1β* expression (Adonis p = 0.02). (**J**) Stacked bar plot of percent relative abundance of taxonomic units within above and below average qPCR log_2_ fold change *IL-1β* expression. Alpha diversity p-values calculated from Mann-Whitney U test between above and below average samples. Beta diversity p-value from Adonis test. Benjamini-Hochberg adjusted Shannon diversity p-value was calculated between the Shannon Diversity, Species Evenness, and Number of Observed Taxonomic Unit metrics together.

There were no significant difference in the Shannon diversity or beta diversity between vagitypes with above-and below-average log_2_ fold *IL-1β* expression when all samples (n =123) were analyzed with DESeq2 (Table 3, **Supplementary Figure 3, Supplementary Figure 4**). However, when stratified into *Lactobacillus*-dominant vagitypes, above-average log_2_ fold change *IL-1β Lactobacillus*-dominant vagitypes exhibited significantly higher Shannon diversity compared to below-average log_2_ fold change *IL-1β Lactobacillus*-dominant vagitypes (Mann Whitney U p-value < 0.05) (Figure 7A–C, Table 3). There was no significant difference in beta diversity between log_2_ fold change *IL-1β* expression across all vagitypes or when partitioning the samples into *Lactobacillus*-dominant vagitypes for the DESeq2 data (Figure 7D, Figure 7I, Table 3, **Figure S3D**).

Similarly, in the qRT-PCR data, there was significantly higher Shannon and beta diversity in the above-average log_2_ fold change of *IL-1β* in participants with *Lactobacillus*-dominant vagitypes (Table 3, Figure 7 F–H). This trend of higher Shannon diversity in samples with above average log_2_ fold change of *IL-1β* was observed when all vagitypes within the qRT-PCR data were compared, though only marginally significantly (Table 3, **Supplementary Figure 4**). Taken together, these results support a hypothesis that, within *Lactobacillus*-dominant vaginal microbiomes, increased microbial Shannon diversity is associated with elevated vaginal proinflammatory *IL-1β* expression.

### Microbial Diversity is Associated with Above Average IL-1β Soluble Cytokine Levels

**Figure 8.**
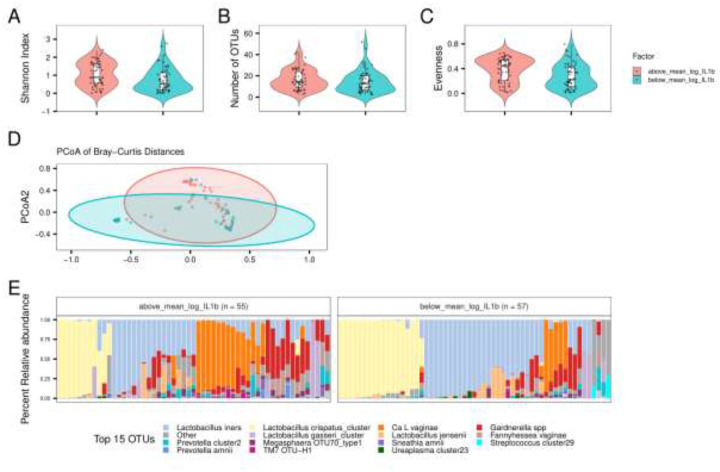
Alpha and Beta Diversity Between Samples with Above and Below Average ln(IL-1β) Soluble Cytokine (**A**)Violin plot of the Shannon diversity between all vagitypes with above and below average ln(IL-1β pg/ mg total protein) n = 112 (p = 0.005. BH-adj p = 0.007). (**B**) Violin plot of the observed taxonomic units between all vagitypes with above and below average ln(IL-1β pg/mg total protein) (p = 0.130, BH-adj p = 0.125). (**C**) Violin plot of the evenness between all vagitypes with above and below average ln(IL-1β pg/mg total protein) (p = 0.003, BH-adj p = 0.007). (**D**) Principal component plot of Bray-Curtis distances between all vagitypes with above and below average ln(IL-1β pg/mg total protein) (*p_adj_ = 0.01). (**E**) Stacked bar plot of all vagitype percent relative abundance of taxonomic units within above and below average ln(IL-1β pg/mg total protein). n =122/123 (99.2%) participants had previously extracted protein and 112/123 (91.1%) participants had IL-1β concentrations within the range of detection of the instrument. Benjamini-Hochberg adjusted Shannon diversity p-value was calculated between the Shannon Diversity, Species Evenness, and Number of Observed Taxonomic Unit metrics together.

To compare IL-1β cytokine protein to DESeq2 gene expression values, soluble IL-1β levels were analyzed from vaginal swabs collected at the same time as the swabs used for transcriptomic and 16S rRNA gene amplicon sequencing. Out of 123 participants, 99.2% (122/123) had cytokine quantification performed previously as described [13], and of those, 10 (8.2%) had a concentration of IL-1β out of range of detection of the instrument and were therefore removed from analysis. There was a significant, positive linear correlation between the DESeq2 *IL-1β* log_2_ fold change value and the natural log-transformed ratio of picograms of IL-1β/milligrams of total sample protein in samples with both datapoints (n = 112) (Spearman’s p-value = 3.8e-7, ρ = 0.46, Pearson’s product-moment correlation p-value = 6.19e-8, ρ = 0.48). Of the samples with detectable IL-1β, the Shannon diversity of samples with above-average ln(pg of IL-1β/mg of total protein) was significantly higher than samples with below-average ln(pg of IL-1β/mg of total protein) (Table 4, Figure 8A–C). The beta diversity was similarly significantly higher in participant samples with above-average ln(pg IL-1β/mg protein) (Table 4, Figure 8D).

The Shannon diversity trended higher in samples with above average ln(pg of IL-1β/mg total protein) when the vagitypes with cytokine measurements were subset to *Lactobacillus*-dominant vagitypes (Figure 8A), but neither the Shannon nor beta diversity values were statistically significant. In summary, samples with above-average IL-1β cytokine expression had significantly higher alpha diversity than samples with below-average IL-1β expression when all vagitypes were analyzed. This indicates that a more complex vaginal microbiome was associated with higher vaginal soluble cytokine concentration, reflecting concurrence between the cytokine and gene expression datasets.

## Discussion

Vaginal and cervical microbiomes with high relative abundance of Gram-positive *Lactobacillus* species are associated with cervicovaginal health. Black and Hispanic women more frequently harbor non-*Lactobacillus*-dominant microbiomes [47], though the extent to which this reflects biological factors versus social determinants of health (including socioeconomic status, healthcare access, and environmental factors) remains unclear. Our host transcriptomic analyses revealed distinct host vaginal gene expression profiles in women with BV-associated vagitypes compared to women with *L. crispatus* dominant vaginal microbiomes in a racioethnically diverse pregnant cohort. Notably, participants with *L. iners* vagitypes, the most common vagitype in our cohort (37.4%), exhibited minimal differential gene expression when compared to participants with *L. crispatus* vagitypes, regardless of racioethnicity (Figure 3, **Figure S2**). The lack of a robust inflammatory transcriptional response observed in the *L. iners* vagitype compared to the BV-associated vagitypes suggests a more nuanced host-microbiome interaction (Figure 3). Similarly, our observation that higher Shannon diversity correlates with elevated vaginal *IL-1β* gene expression, particularly within *Lactobacillus*-dominant vagitypes, suggests that even modest increases in microbial diversity may influence host inflammatory transcriptional responses when *Lactobacillus* species remain predominant. The mechanisms underlying this diversity/inflammation relationship warrant further investigation, particularly to address whether specific subdominant taxa drive inflammatory responses or whether microbiome diversity itself alters *Lactobacillus* metabolic activity in ways that affect host immunity. Taken together, these findings support the characterization of *L. iners* as an intermediate state between an optimal and dysbiotic vaginal microbiome [6].

These RNA-seq results were, generally, directionally consistent with the expression of four immune/inflammatory genes (*IL-1β, NCF2, CXCL8, and BCL2A1*) via qRT-PCR. We additionally observed higher Shannon diversity in samples with above-average expression of IL-1β at both the RNA and protein levels. Because the data presented here are cross-sectional, we cannot determine causality: microbiome composition may drive host inflammatory gene expression, a pre-existing host inflammatory state may drive microbiome composition, or both may occur. Longitudinal studies tracking microbiome composition and host gene expression over time will be essential to disentangle these relationships. Nevertheless, *in vitro* and *in vivo* studies demonstrate increased inflammatory cytokine mRNA and protein in the cervicovaginal environment with exposure to BV-associated microbiome taxa [11, 17–26, 28, 30–39, 84, 89, 90]. Taken together, our results indicate an association between the *Ca*. L. vaginae, *Gardnerella* spp., and the No Type vagitypes with increased local vaginal inflammatory gene and protein expression in pregnant women when compared to participants with *L. crispatus* dominant vaginal microbiomes.

Bacteria drivers of host inflammatory cytokine gene and protein expression in the cervicovaginal cavity include, but are not limited to, secretion systems and surface proteins such as toxins [91]. *Gardnerella* spp. and *Sneathia vaginalis* both produce pore forming toxins, with *Gardnerella* spp. producing the cholesterol dependent cytolysin vaginolysin and *S. vaginalis* producing cytopathogenic toxin, component A (CptA) [92]. Vaginolysin has been shown to elicit proinflammatory cytokine expression and epithelial cell damage *in vitro* [93], and CptA causes cell permeability and red blood cell lysis [92]. *In vitro* studies have shown that *Gardnerella* spp. vaginolysin is expressed in the presence of *Lactobacillus* species [94] and that vaginolysin expression is significantly higher in planktonic *Gardnerella* compared to *Gardnerella* within biofilms [95]. In heterogenous microbiomes (~50% ≥ relative abundance of *Lactobacillus*), *Gardnerella* may persist and continue to produce vaginolysin—escaping both *Lactobacillus* suppression and biofilm restraint—thereby modulating host *IL-1β* expression. Additionally, some strains of *Gardnerella vaginalis* can both stimulate TLR-2 and compete with *Lactobacillus* spp. for the anti-inflammatory receptor DC-SIGN, which could contribute to an altered immune state in a *Lactobacillus*-dominant vaginal microbiome with a large subpopulation of *Gardnerella* [96]. These hypothetical dynamics are notable as *Gardnerella* spp. commonly co-exist with high relative abundance of *L. iners* in the vaginal microbiome [6]. Future mechanistic experiments employing *in vitro* models with mixed microbial communities will be critical for elucidating the precise mechanisms and community compositions by which these taxa modulate host inflammation.

Reduced expression of *CSTB*, an inhibitor of the NLRP3 inflammasome [74, 75], in all three BV-associated vagitypes could indicate a potential method of dysregulation of host cell mitochondrial function and increased NLRP3 activation among anaerobic cervicovaginal taxa. Both CSTB and CSTA proteins were shown to be downregulated in participants with diverse cervicovaginal microbiome communities in non-pregnant Kenyan women compared to those with *Lactobacillus*-dominant microbiomes [37]. Primary bone marrow-derived macrophages from CSTB knockout mice secreted higher levels of IL-1β and TNF-α, had increased mitochondrial damage and superoxide generation, and had increased caspase activation after lipopolysaccharide challenge [75]. Multiple PAMPs and damage associated molecular patterns (DAMPs), including mitochondrial reactive oxygen species and extracellular adenosine triphosphate (ATP), can lead to NLRP3 inflammasome activation and subsequent cleavage of *pro-IL-1β* into mature *IL-1β* [97]. *Gardnerella* spp. induces NLRP3 inflammasomes in THP-1 monocyte-like cells [34, 35] and primary mouse macrophages *in vitro* [35]. The mechanisms by which BV-associated taxa could downregulate *CSTB* to facilitate NLRP3 activation and increase oxidative stress are unknown. Repression of CSTB mRNA/protein by the pore-forming toxin listeriolysin O [98] and by *Salmonella typhimurium* kinase T4519 [99] –the latter suppressing *CSTB* mRNA expression through interaction with toll-like receptor 2, which *Gardnerella* spp. also engages [96] –supports a potential bacterial effector-driven mechanism. In addition to potential toxin-driven downregulation of *CSTB* contributing to NLRP3 activation, bacterial pore-forming toxin-derived disruption of intracellular calcium and potassium gradients, including but not limited to listeriolysin*, Staphylococcus aureus* alpha toxin, and aerolysin from *Aeromonas hydrophila*, directly drive NLRP3 inflammasome activation [100].

Similarities in the activation of inflammasomes in infectious cervicovaginal diseases, bacterial vaginosis, preterm birth, and chorioamnionitis warrants further investigation. *Candida* spp.*, Trichomonas vaginalis, Chlamydia trachomatis*, and *Neisseria gonorrhea* infections have all demonstrated NLRP3 inflammasome activation and proinflammatory cytokine secretion [101–109]. Endometritis is associated with both bacterial vaginosis associated organisms [14], as well as increased NLRP3 activation and inflammatory cytokine production in murine models [110, 111]. Romero et al. have demonstrated increased NLRP3 activation and secretion of mature IL-1β in both women who delivered preterm or at term with chorioamnionitis/pathologic placental lesions [112–115]. As inflammasomes have been shown to play a crucial role in epithelial maintenance and microbiome colonization in the gut [116], their analogous role in vaginal epithelium warrants investigation. They are upregulated in perinatal tissue during term labor without infection [117], suggesting a delicate balance between protective and deleterious NLRP3 activation. *Gardnerella*, the bacterium most often associated with bacterial vaginosis [118], has been shown to activate the NLRP3 inflammasome *in vitro* [34, 35], though NLRP3 inflammasome activation in the cervicovaginal epithelium in concordance with bacterial vaginosis status has not been examined *in vivo*. Future research will clarify NLRP3 inflammasome component expression in cervicovaginal tissue and/or cells of women with BV compared to women without BV. Likewise, future studies should characterize inflammasomes in perinatal tissues in term and preterm births with and without chorioamnionitis pathology alongside cervicovaginal microbiome 16S rRNA gene amplicon sequencing to assess their relationship.

The main limitation of this study is the confounding of the *Gardnerella* spp. and *Ca*. L. vaginae vagitypes with self-reported racioethnicity in the DESeq2 data. The qRT-PCR participants exhibited slightly greater racial diversity within *Gardnerella* spp. vagitypes **(Supplementary table 8)** than the DESeq2 participants. To address this, the baseline *L. crispatus* vagitype group used for comparison in the DESeq2 and qRT-PCR data had representation of all racioethnicities. However, the observation that BV-associated vagitypes also exhibited distinct gene expression profiles relative to *L. crispatus* vagitypes, even when metatranscriptomic analysis was restricted to Black participants (**Figure S2**), suggests that racioethnicity alone does not account for the distinct gene expression profiles observed between vagitypes. A further consideration is that the majority (69.9%) of Black participants in this study reported an annual income of < $15,000, while 78.6% of the White participants reported an annual income of > $15,000. Given evidence that financial insecurity affects gynecological health and pregnancy outcomes [119–121], as well as general inflammation [122], the significantly expressed immune response genes in the BV-associated vagitypes compared to the *L. crispatus* vagitype cannot be completely disentangled from the effects of racioethnicity and socioeconomic status.

A second limitation of this study is the paucity of information of the HPV vaccination status of the participants. Without participant vaccination records or the performance of the HPV PCR screening for every sample derived from each patient, a substantial portion of participants lack HPV vaccination history and HPV infection status during their pregnancy. Similarly, the questionnaire phrasing did not require patients to distinguish if they received the Gardasil^®^ or Cerverix^®^ vaccine. There is evidence of differing protective immunity induced by the two vaccines including differing immunoglobulin titers and protection against adenocarcinoma and carcinoma [123, 124]. It has been shown that HPV infection and cervicovaginal microbiome diversity are intertwined [61, 125, 126], necessitating more comprehensive HPV infection screening and acquisition of vaccination records for future host transcriptomic studies.

The final limitation of this study is a lack of vaginal transcriptomic samples from non-pregnant women with a similar distribution of racioethnicities. While the analysis presented here identifies genes significantly expressed between vagitypes within a pregnant cohort, the same genes may not be differentially regulated in non-pregnant women. It is likely that the heightened immune state of pregnancy [127] has unique effects on the host vaginal transcriptome that might not be recapitulated in that of non-pregnant women with similar demographics.

## Conclusion

In summary, we demonstrate that the host transcriptome during pregnancy is associated with vaginal microbiome diversity. Participants with BV-associated vagitypes had unique vaginal transcriptional profiles from those with *Lactobacillus crispatus*-dominant vaginal microbiomes, including multiple differentially expressed genes associated within immune response pathways and cell maintenance. Our present study emphasizes the importance of accounting for host demographic factors such as self-reported racioethnicity and socioeconomic status in dual microbiome/host transcriptome analysis during pregnancy, while demonstrating that vagitype-associated transcriptional differences persist independent of racioethnicity. These results also reveal a potential link between cervicovaginal bacterial effector proteins and host NLRP3 inflammasome activation that warrants further investigation.

## Supplementary Material

Supplementary Files

This is a list of supplementary files associated with this preprint. Click to download.

- OneDrive13302026.zip

## Figures and Tables

**Table 1.** Description of Participant Demographics for Host Gene Differential Expression Analysis with DESeq2

	Participant Self-Reported Racioethnicity			
n_total_ = 123	Black (n = 83, 67.5%)	White (n = 28, 22.8%)	Mixed Racioethnicity (n = 7, 5.7%)	Hispanic or Latina (n = 5, 4.1%)
**Maternal Age**				
Mean (SD), 27.46 (± 5.66)	26.56 (± 5.62)	30.4 (± 5.0)	25.7 (± 6.78)	28 (± 3.0)
Median (IQR), 27 (± 8.0)	26.0 (± 7.0)	30.0 (± 3.5)	23.0 (± 9.0)	28 (± 3.0)
**Birth Status**				
Delivered Preterm (33, 26.8%)	23 (27.7%)	7 (25%)	1 (16.7%)	2 (66.6%)
Delivered at Term (90, 73.2%)	60 (72.3%)	21 (75%)	6 (85.7%)	3 (33.3%)
**HPV Status**				
HPV positive (42, 34.1%)	27 (32.5%)	8 (28.6%)	5 (71.4%)	2 (40%)
HPV negative (56, 44.7%)	38 (45.8%)	14 (5%)	1 (14.3%)	3 (60%)
Unknown (25, 20.3%)	18 (21.7%)	6 (21.4%)	1 (14.3%)	0
**HPV Vaccination Status**				
Vaccinated (Gardasil^®^ or Cerverix^®^) (35, 28.5%)	22 (26.5%)	7 (25%)	4 (5.7%)	2 (40%)
Unvaccinated (71, 57.7%)	48 (57.8%)	19 (67.9%)	2 (28.6%)	2 (40%)
Not sure (17, 13.8%)	13 (15.7%)	2 (7.1%)	1 (14.3%)	1 (20%)
**Sample Collection Gestational Age in weeks**				
Mean (SD), 174.0 (± 50.7)	167.8 (± 51.3)	184.6 (± 41.1)	177.4 (± 75.4)	182 (± 42.6)
Median (IQR), 168 (± 81)	152 (± 82.0)	192.5 (± 63.8)	203 (± 86.5)	179 (± 42.5)
**Income**				
Annual Income > $15,000 (46, 37.4%)	20 (24.1%)	22 (78.6%)	3 (42.9%)	1 (20%)
Annual Income < $15,000 (71, 57.7%)	58 (69.9 %)	6 (21. 4%)	3 (42.9%)	4 (80%)
Unknown Annual Income (6, 4.9%)	5 (6.02 %)	0	1 (14.3%)	0
**Medication Taken at the time of Sampling**				
No Medication Reported (109, 88.6%)	76 (91.6%)	22 (78.6%)	6 (85.7%)	5 (100%)
Non-infectious medication (12,9.7%)	5 (6.02%)	6 (21.4%)	1 (14.3%)	0
Progesterone (2, 1.6%)	2 (2.4%)	0	0	0
**Systolic Blood Pressure**				
Mean (SD), 115.1 (± 11.6)	116.4 (± 11.54)	112.4 (±12.1)	113.9 (± 12.2)	108.7 (± 3.1)
Median (IQR), 113.0 (± 18.0)	115 (±17.0)	110.0 (±18.75)	111 (± 17.5)	108.0 (± 3)
***Lactobacillus crispatus cluster* vagitype** (n = 30, 24.4%)	13 (43.3%)	14 (46.7%)	1 (3.3%)	2 (6.7%)
***Gardnerella* spp. vagitype** (n =14, 11.4%)	14 (100%)	0	0	0
***Ca*. Lachnocurva vaginae vagitype** (n = 22, 17.9%)	18 (81.8%)	2 (9.1%)	2 (9.1%)	0
***Lactobacillus gasseri cluster* vagitype** (n = 4, 3.3%)	3 (75%)	0	0	1 (25%)
***Lactobacillus iners* vagitype** (n = 46, 37.4%)	32 (69.6%)	9 (19.6%)	4 (8.7%)	1 (2.2%)
**No Type vagitype** (n = 7, 5.7%)	3 (42.9%)	2 (28.6%)	0	2 (28.6%)

**Table 2.** Differentially Regulated Inflammasome and NF-kB Pathway Genes in BV-Associated Vagitypes Compared to *L. crispatus*

Gene Name	Significant Vagitype Contrast	BH adj p-value	DESeq2 Fold Change Direction	Literature
*IL-1β*	*Ca*. L. vaginae *vs. L. crispatus*	0.01771	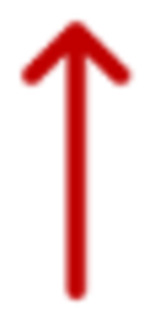	[68]
*NLRP3*	*Ca*. L. vaginae *vs. L. crispatus*	0.022935	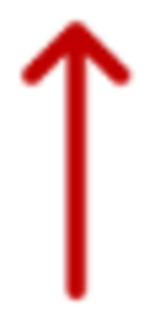	[68]
*GBP5*	*Ca*. L. vaginae *vs. L. crispatus*	0.033364	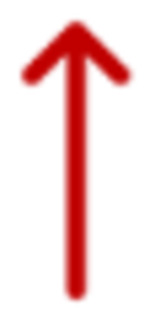	[69]
*TICAM1*	*Ca*. L. vaginae *vs. L. crispatus, Gardnerella* spp. *vs. L. crispatus, No Type vs. L. crispatus*	0.008482034, 0.00043607, 8.88E-07	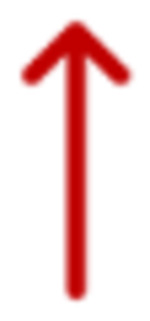	[70]
*INAVA*	*Ca*. L. vaginae *vs. L. crispatus, Gardnerella* spp. *vs. L. crispatus, No Type vs. L. crispatus*	2.41E-05, 0.001919455, 0.022489143	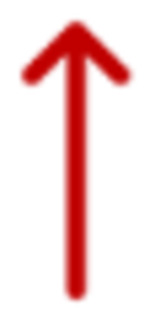	[71]
*JUN*	*Ca*. L. vaginae *vs. L. crispatus, Gardnerella* spp. *vs. L. crispatus, No Type vs. L. crispatus*	0.000379335, 0.000482357, 0.00015186	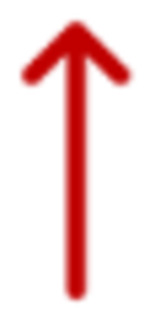	[72, 73]
*CSTB*	*Ca*. L. vaginae *vs. L. crispatus, Gardnerella* spp. *vs. L. crispatus, No Type vs. L. crispatus*	0.005945427, 0.003493309, 3.22E-05	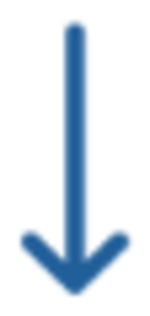	[74, 75]

**Table 3.** Measures of Central Tendency of the log_2_ fold change of *IL-1β* and Microbiome Diversity Metrics

Dataset	Dataset Description	Mean *IL-1B* |LOG2FC| (± S.D)	Median *IL-1B* |LOG2FC| (IQR)	Shannon diversity test p-value	Shannon test BH adjusted p-value	Beta Diversity Adonis Test P-Value
**DESEQ2 DATA**	All vagitypes together (n =123)	1.83 (± 1.20)	1.59 (± 1.88)	0.374 (Effect size = 0.08 Cliff’s Delta = 0.09)	0.535	0.40
**DESEQ2 DATA**	*L. crispatus, L. iners*, and *L. gasseri* vagitypes (n = 80)	1.82 (± 1.12)	1.61 (± 1.80)	0.047 (Effect size = 0.22) Cliff’s Delta = 0.26)	0.071	0.41
**qRT-PCR DATA**	All vagitypes together (n = 87)**	1.62 (± 1.21)	1.31 (± 1.66)	0.051 (Effect size = 0.209 Cliff’s Delta = 0.244)	0.086	0.100
**qRT-PCR DATA**	*L. crispatus, L. iners*, and *L. gasseri* vagitypes (n= 59)**	1.28 (± 0.98)	1.12 (± 1.36)	0.0004 (Effect size = 0.447) (Cliff’s Delta 0.52)	0.001	0.002

* Benjamini-Hochberg adjusted Shannon diversity Mann-Whitney U test p-values calculated between the Shannon Diversity, Species Evenness, and Number of Observed Taxonomic Unit together.

** Not all samples analyzed with qRT-PCR had detectable IL-1β expression

**Table 4.** Measures of Central Tendency of ln(pg IL-1β/mg of total protein) and Microbiome Diversity Metrics

Dataset	Dataset description	Mean ln(pg of IL-1β/mg total protein) (± SD)	Median ln(pg of Il-1β/mg total protein) (IQR)	Shannon diversity test p-value	Shannon diversity test BH adjusted p-value *	Beta diversity adonis test p-value
**Cytokine Data from Deseq2 Participants**	All vagitypes together (n =112)	5.57 (± 1.45)	6.61 (2.03)	0.005 (Effect size = 0.266) (Cliff’s Delta = 0.309)	0.007	0.01
**Cytokine Data from Deseq2 Participants**	*Lactobacillus*-dominant vagitypes (n = 75)	5.22 (± 1.38)	5.17 (1.98)	0.173 (Effect size = 0.159) (Cliff’s Delta = 0.186)	0.255	0.334

* Benjamini-Hochberg adjusted Shannon diversity Mann-Whitney U test p-values calculated between the Shannon Diversity, Species Evenness, and Number of Observed Taxonomic Unit together.

## Data Availability

The datasets supporting the conclusions of this article are available in the Database of Genotypes and Phenotypes (dbGaP) (study no. 20280; accession ID phs001523.v1.p1), and the SRA under BioProject PRJNA326441.

## Abbreviations

NLRP3: NLR family pyrin domain containing 3
STI: sexually transmitted infections
HIV: Human immunodeficiency virus
MOMS-PI: Multi-Omic Microbiome Study-Pregnancy Initiative
rRNA: ribosomal ribonucleic acid
qRT-PCR: quantitative polymerase chain reaction
BV: bacterial vaginosis
TLR-2: Toll-like receptor 2
PCA: principal component analysis
VCU: Virginia Commonwealth University

## References

[1] Huttenhower C, Gevers D, Knight R, Abubucker S, Badger JH, Chinwalla AT, Structure, function and diversity of the healthy human microbiome. Nature. 2012;486:207–14. 10.1038/nature11234. 22699609 PMC3564958

[2] Gupta P, Singh MP, Goyal K. Diversity of Vaginal Microbiome in Pregnancy: Deciphering the Obscurity. Front Public Health. 2020;8. 10.3389/fpubh.2020.00326. PMC7393601 32793540

[3] Lehtoranta L, Ala-Jaakkola R, Laitila A, Maukonen J. Healthy Vaginal Microbiota and Influence of Probiotics Across the Female Life Span. Front Microbiol. 2022;13. 10.3389/fmicb.2022.819958. PMC9024219 35464937

[4] Delgado-Diaz DJ, Tyssen D, Hayward JA, Gugasyan R, Hearps AC, Tachedjian G. Distinct Immune Responses Elicited From Cervicovaginal Epithelial Cells by Lactic Acid and Short Chain Fatty Acids Associated With Optimal and Non-optimal Vaginal Microbiota. Front Cell Infect Microbiol. 2020;9. 10.3389/fcimb.2019.00446. PMC6965070 31998660

[5] Witkin SS, Mendes-Soares H, Linhares IM, Jayaram A, Ledger WJ, Forney LJ. Influence of vaginal bacteria and D- and L-lactic acid isomers on vaginal extracellular matrix metalloproteinase inducer: Implications for protection against upper genital tract infections. mBio. 2013;4. 10.1128/mBio.00460-13. PMC3735189 23919998

[6] Verstraelen H, Verhelst R, Claeys G, De Backer E, Temmerman M, Vaneechoutte M. Longitudinal analysis of the vaginal microflora in pregnancy suggests that L. crispatus promotes the stability of the normal vaginal microflora and that L. gasseri and/or L. iners are more conducive to the occurrence of abnormal vaginal microflora. BMC Microbiol. 2009;9. 10.1186/1471-2180-9-116. PMC2698831 19490622

[7] Walensky R, Houry D, Jernigan D, Bunnell R, Layden J, Iademarco M, Sexually Transmitted Infections Treatment Guidelines, 2021, Morbidity and Mortality Weekly Report--Bacterial Vaginosis. 2021.

[8] Koumans EH, Sternberg M, Bruce C, McQuillan G, Kendrick J, Sutton M, The prevalence of bacterial vaginosis in the United States, 2001–2004; associations with symptoms, sexual behaviors, and reproductive health. Sex Transm Dis. 2007;34:864–9. 10.1097/OLQ.0b013e318074e565. 17621244

[9] Peebles K, Velloza J, Balkus JE, McClelland RS, Barnabas R V. High Global Burden and Costs of Bacterial Vaginosis: A Systematic Review and Meta-Analysis. Sex Transm Dis. 2019;46:304–11. 10.1097/OLQ.0000000000000972. 30624309

[10] Bradshaw CS, Morton AN, Hocking J, Garland SM, Morris MB, Moss LM, High Recurrence Rates of Bacterial Vaginosis over the Course of 12 Months after Oral Metronidazole Therapy and Factors Associated with Recurrence. J Infect Dis. 2006;:193. 10.1086/503780. 16652274

[11] Masson L, Mlisana K, Little F, Werner L, Mkhize NN, Ronacher K, Defining genital tract cytokine signatures of sexually transmitted infections and bacterial vaginosis in women at high risk of HIV infection: A cross-sectional study. Sex Transm Infect. 2014;90:580–7. 10.1136/sextrans-2014-051601. 25107710

[12] Elovitz MA, Gajer P, Riis V, Brown AG, Humphrys MS, Holm JB, Cervicovaginal microbiota and local immune response modulate the risk of spontaneous preterm delivery. Nat Commun. 2019;10. 10.1038/s41467-019-09285-9. PMC6428888 30899005

[13] Fettweis JM, Serrano MG, Brooks JP, Edwards DJ, Girerd PH, Parikh HI, The vaginal microbiome and preterm birth. Nat Med. 2019;25:1012–21. 10.1038/s41591-019-0450-2. 31142849 PMC6750801

[14] Ravel J, Moreno I, Simón C. Bacterial vaginosis and its association with infertility, endometritis, and pelvic inflammatory disease. Am J Obstet Gynecol. 2021;224:251–7. 10.1016/j.ajog.2020.10.019. 33091407

[15] Alcaide ML, Strbo N, Romero L, Jones DL, Rodriguez VJ, Arheart K, Bacterial vaginosis is associated with loss of gamma delta T cells in the female reproductive tract in women in the Miami Women Interagency HIV Study (WIHS): A cross sectional study. PLoS One. 2016;11. 10.1371/journal.pone.0153045. PMC4831836 27078021

[16] Thurman AR, Kimble T, Herold B, Mesquita PMM, Fichorova RN, Dawood HY, Bacterial Vaginosis and Subclinical Markers of Genital Tract Inflammation and Mucosal Immunity. AIDS Res Hum Retroviruses. 2015;31:1139–52. 10.1089/aid.2015.0006. 26204200 PMC4651020

[17] Eade CR, Diaz C, Wood MP, Anastos K, Patterson BK, Gupta P, Identification and Characterization of Bacterial Vaginosis-Associated Pathogens Using a Comprehensive Cervical-Vaginal Epithelial Coculture Assay. PLoS One. 2012;7. 10.1371/journal.pone.0050106. PMC3499514 23166828

[18] Cauci S, Guaschino S, Driussi S, De Santo D, Lanzafame P, Quadrifoglio F. Correlation of Local Interleukin-8 with Immunoglobulin A against Gardnerella vaginalis Hemolysin and with Prolidase and Sialidase Levels in Women with Bacterial Vaginosis. J Infect Dis. 2002;11:185–1620. 10.1086/340417. 12023767

[19] Anahtar MN, Byrne EH, Doherty KE, Bowman BA, Yamamoto HS, Soumillon M, Cervicovaginal Bacteria Are a Major Modulator of Host Inflammatory Responses in the Female Genital Tract. Immunity. 2015;42:965–76. 10.1016/j.immuni.2015.04.019. 25992865 PMC4461369

[20] Masson L, Barnabas S, Deese J, Lennard K, Dabee S, Gamieldien H, Inflammatory cytokine biomarkers of asymptomatic sexually transmitted infections and vaginal dysbiosis: A multicentre validation study. Sex Transm Infect. 2019;95:5–12. 10.1136/sextrans-2017-053506. 30018088

[21] Rebbapragada A, Howe K, Wachihi C, Pettengell C, Sunderji S, Huibner S, Bacterial Vaginosis in HIV-Infected Women Induces Reversible Alterations in the Cervical Immune Environment. J Acquir Immune Defic Syndr (1988). 2008;49. 10.1097/qai.0b013e318189a7ca. 18989228

[22] Cauci S, Guaschino S, De Aloysio D, Driussi S, De Santo D, Penacchioni P, Interrelationships of interleukin-8 with interleukin-1b and neutrophils in vaginal fluid of healthy and bacterial vaginosis positive women. Mol Hum Reprod. 2003;9:53–8. 10.1093/humrep/gag003. 12529421

[23] MacLean F, Tsegaye AT, Graham JB, Swarts JL, Vick SC, Potchen NB, Bacterial vaginosis associates with dysfunctional T cells and altered soluble immune factors in the cervicovaginal tract. J Clin Invest. 2025;135. 10.1172/JCI184609. PMC12077898 40131862

[24] Reza Zariffard M, Novak RM, Lurain N, Sha BE, Graham P, Spear GT. Induction of Tumor Necrosis Factor-a Secretion and Toll-Like Receptor 2 and 4 mRNA Expression by Genital Mucosal Fluids from Women with Bacterial Vaginosis. J Infect Dis. 2005;191:1913–21. 10.1086/429922. 15871126

[25] Mares D, Simoes JA, Novak RM, Spear GT. TLR2-mediated cell stimulation in bacterial vaginosis. J Reprod Immunol. 2008;77:91–9. 10.1016/j.jri.2007.04.004. 17532476 PMC2254576

[26] Borgdorff H, Gautam R, Armstrong SD, Xia D, Ndayisaba GF, Van Teijlingen NH, Cervicovaginal microbiome dysbiosis is associated with proteome changes related to alterations of the cervicovaginal mucosal barrier. Mucosal Immunol. 2016;9:621–33. 10.1038/mi.2015.86. 26349657

[27] Sakai M, Ishiyama A, Tabata M, Sasaki Y, Yoneda S, Shiozaki A, Relationship between cervical mucus interleukin-8 concentrations and vaginal bacteria in pregnancy. American Journal of Reproductive Immunology. 2004;52:106–12. 10.1111/j.1600-0897.2004.00203.x. 15274649

[28] Anton L, Ferguson B, Friedman ES, Gerson KD, Brown AG, Elovitz MA. Gardnerella vaginalis alters cervicovaginal epithelial cell function through microbe-specific immune responses. Microbiome. 2022;10. 10.1186/s40168-022-01317-9. PMC9351251 35922830

[29] Jean S, Huang B, Parikh HI, Edwards DJ, Brooks JP, Kumar NG, Multi-omic Microbiome Profiles in the Female Reproductive Tract in Early Pregnancy. Infectious Microbes and Diseases. 2019;1:49–60. 10.1097/IM9.0000000000000007.

[30] Price JT, Vwalika B, France M, Ravel J, Ma B, Mwape H, HIV-associated vaginal microbiome and inflammation predict spontaneous preterm birth in Zambia. Sci Rep. 2022;12. 10.1038/s41598-022-12424-w. PMC9123167 35595739

[31] Libby EK, Pascal KE, Mordechai E, Adelson ME, Trama JP. Atopobium vaginae triggers an innate immune response in an in vitro model of bacterial vaginosis. Microbes Infect. 2008;10:439–46. 10.1016/j.micinf.2008.01.004. 18403235

[32] Zalenskaya IA, Joseph T, Bavarva J, Yousefieh N, Jackson SS, Fashemi T, Gene expression profiling of human vaginal cells in vitro discriminates compounds with pro-inflammatory and mucosa-altering properties: Novel biomarkers for preclinical testing of HIV microbicide candidates. PLoS One. 2015;10. 10.1371/journal.pone.0128557. PMC4459878 26052926

[33] Fichorova RN, Yamamoto HS, Delaney ML, Onderdonk AB, Doncel GF. Novel vaginal microflora colonization model providing new insight into microbicide mechanism of action. mBio. 2011;2. 10.1128/mBio.00168-11. PMC3202752 22027006

[34] Vick EJ, Park HS, Huff KA, Brooks KM, Farone AL, Farone MB. Gardnerella vaginalis triggers NLRP3 inflammasome recruitment in THP-1 monocytes. J Reprod Immunol. 2014;106:67–75. 10.1016/j.jri.2014.08.005. 25280956

[35] Xiang N, Yin T, Chen T. Gardnerella vaginalis induces NLRP3 inflammasome-mediated pyroptosis in macrophages and THP-1 monocytes. Exp Ther Med. 2021;22. 10.3892/etm.2021.10609. PMC8393845 34504619

[36] Byrne EH, Farcasanu M, Bloom SM, Xulu N, Xu J, Hykes BL, Antigen Presenting Cells Link the Female Genital Tract Microbiome to Mucosal Inflammation, With Hormonal Contraception as an Additional Modulator of Inflammatory Signatures. Front Cell Infect Microbiol. 2021;11. 10.3389/fcimb.2021.733619. PMC8482842 34604114

[37] Edfeldt G, Kaldhusdal V, Czarnewski P, Bradley F, Bergström S, Lajoie J, Distinct cervical tissue-adherent and luminal microbiome communities correlate with mucosal host gene expression and protein levels in Kenyan sex workers. Microbiome. 2023;11. 10.1186/s40168-023-01502-4. PMC10064689 37004130

[38] Cheng L, Ka D, Norenhag J, Hamsten M, Fransson E, Schuppe-Koistinen I, A MicroRNA Gene Panel Predicts the Vaginal Microbiota Composition. mSystems. 2021;6. 10.1128/mSystems. PMC8269211 33947805

[39] Berard AR, Brubaker DK, Birse K, Lamont A, Mackelprang RD, Noël-Romas L, Vaginal epithelial dysfunction is mediated by the microbiome, metabolome, and mTOR signaling. Cell Rep. 2023;42. 10.1016/j.celrep.2023.112474. PMC10242450 37149863

[40] Underhill LA, Mennella JM, Tollefson GA, Uzun A, Lechner BE. Transcriptomic analysis delineates preterm prelabor rupture of membranes from preterm labor in preterm fetal membranes. BMC Med Genomics. 2024;17. 10.1186/s12920-024-01841-7. PMC10916314 38443884

[41] Tarca AL, Pataki BÁ, Romero R, Sirota M, Guan Y, Kutum R, Crowdsourcing assessment of maternal blood multi-omics for predicting gestational age and preterm birth. Cell Rep Med. 2021;2. 10.1016/j.xcrm.2021.100323. PMC8233692 34195686

[42] Romero R, Mazor M. Infection and Preterm Labor. Clin Obstet Gynecol. 1988;31. 10.1097/00003081-198809000-00006. 3066544

[43] Molina B. Cervicovaginal inflammation and neutrophil infiltration/activation in women at high-risk of prematurity. BJOG. 2022;129:47–62. 10.1111/1471-0528.8_17178.

[44] Chambers S, Pons JC, Richard A, Chiesa M, Bouyer J, Papiemik E. Vaginal infections, cervical ripening and preterm delivery. European Journal of Obstetrics and Gynecology and Reproductive Biology. 1990;38:103–8. 10.1016/0028-2243(91)90185-n. 1995377

[45] Wikström T, Abrahamsson S, Bengtsson-Palme J, Ek J, Kuusela P, Rekabdar E, Microbial and human transcriptome in vaginal fluid at midgestation: Association with spontaneous preterm delivery. Clin Transl Med. 2022;12. 10.1002/ctm2.1023. PMC9473488 36103557

[46] Mohd Zaki A, Hadingham A, Flaviani F, Haque Y, Mi JD, Finucane D, Neutrophils Dominate the Cervical Immune Cell Population in Pregnancy and Their Transcriptome Correlates With the Microbial Vaginal Environment. Front Microbiol. 2022;13. 10.3389/fmicb.2022.904451. PMC9237529 35774454

[47] Gajer P, Brotman RM, Bai G, Sakamoto J, Schütte UME, Zhong X, Temporal dynamics of the human vaginal microbiota. Sci Transl Med. 2012;4. 10.1126/scitranslmed.3003605. PMC3722878 22553250

[48] Serrano MG, Parikh HI, Brooks JP, Edwards DJ, Arodz TJ, Edupuganti L, Racioethnic diversity in the dynamics of the vaginal microbiome during pregnancy. Nat Med. 2019;25:1001–11. 10.1038/s41591-019-0465-8. 31142850 PMC6746180

[49] Fettweis JM, Paul Brooks J, Serrano MG, Sheth NU, Girerd PH, Edwards DJ, Differences in vaginal microbiome in African American women versus women of European ancestry. Microbiology (United Kingdom). 2014;160:2272–82. 10.1099/mic.0.081034-0. PMC4178329 25073854

[50] Fettweis JM, Serrano MG, Sheth NU, Mayer CM, Glascock AL, Brooks JP, Species-level classification of the vaginal microbiome. BMC Genomics. 2012;13 Suppl 8. 10.1186/1471-2164-13-s8-s17. PMC3535711 23282177

[51] Ahmed A, Earl J, Retchless A, Hillier SL, Rabe LK, Cherpes TL, Comparative genomic analyses of 17 clinical isolates of Gardnerella vaginalis provide evidence of multiple genetically isolated clades consistent with subspeciation into genovars. J Bacteriol. 2012;194:3922–37. 10.1128/JB.00056-12. 22609915 PMC3416530

[52] Callahan BJ, DiGiulio DB, Aliaga Goltsman DS, Sun CL, Costello EK, Jeganathan P, Replication and refinement of a vaginal microbial signature of preterm birth in two racially distinct cohorts of US women. Proc Natl Acad Sci U S A. 2017;114:9966–71. 10.1073/pnas.1705899114. 28847941 PMC5604014

[53] Vaneechoutte M, Guschin A, Van Simaey L, Gansemans Y, Van Nieuwerburgh F, Cools P. Emended description of Gardnerella vaginalis and description of gardnerella leopoldii sp. Nov., gardnerella piotii sp. nov. and Gardnerella swidsinskii sp. nov., with delineation of 13 genomic species within the genus Gardnerella. Int J Syst Evol Microbiol. 2019;69:679–87. 10.1099/ijsem.0.003200. 30648938

[54] Chen J, Zhang X, Yang L, Zhang L. GUniFrac: Generalized UniFrac Distances, DistanceBased Multivariate Methods and Feature-Based Univariate Methods for Microbiome Data Analysis. CRAN: Contributed Packages. 2012. 10.32614/CRAN.package.GUniFrac.

[55] Cameron ES, Schmidt PJ, Tremblay BJM, Emelko MB, Müller KM. Enhancing diversity analysis by repeatedly rarefying next generation sequencing data describing microbial communities. Sci Rep. 2021;11. 10.1038/s41598-021-01636-1. PMC8595385 34785722

[56] McMurdie PJ, Holmes S. Phyloseq: An R Package for Reproducible Interactive Analysis and Graphics of Microbiome Census Data. PLoS One. 2013;8. 10.1371/journal.pone.0061217. PMC3632530 23630581

[57] R Core Team and contributors worldwide. STAT: Interactive Document for Working with Basic Statistical Analysis. CRAN: Contributed Packages. 2019. 10.32614/CRAN.package.STAT.

[58] Oksanen J, Simpson GL, Blanchet FG, Kindt R, Legendre P, Minchin PR, vegan: Community Ecology Package. CRAN: Contributed Packages. 2001. 10.32614/CRAN.package.vegan.

[59] Chen S, Zhou Y, Chen Y, Gu J. Fastp: An ultra-fast all-in-one FASTQ preprocessor. Bioinformatics. 2018;34:i884–90. 10.1093/bioinformatics/bty560. 30423086 PMC6129281

[60] Wood DE, Lu J, Langmead B. Improved metagenomic analysis with Kraken 2. Genome Biol. 2019;20. 10.1186/s13059-019-1891-0. PMC6883579 31779668

[61] Bridy P V., Cruz JC, Covington JL, Islam TI, Hadley CE, Tran K, Human papillomavirus 16 mitigates Sneathia vaginalis -induced damage to cervical keratinocytes. mSphere. 2025;10. 10.1128/msphere.00152-25. PMC12306162 40590527

[62] Zhao M, Kang P, Zhu L, Zhou D, Cui M, Zhang M, Global pattern of persistent human papillomavirus infection in female genital tract: An update system review and meta-analysis. iScience. 2024;27. 10.1016/j.isci.2024.110991. PMC11519437 39474077

[63] Love MI, Huber W, Anders S. Moderated estimation of fold change and dispersion for RNA-seq data with DESeq2. Genome Biol. 2014;15. 10.1186/s13059-014-0550-8. PMC4302049 25516281

[64] Carlson M. org.Hs.eg.db: Genome wide annotation for Human. 2025. 10.18129/B9.bioc.org.Hs.eg.db.

[65] Hoffman GE, Schadt EE. variancePartition: Interpreting drivers of variation in complex gene expression studies. BMC Bioinformatics. 2016;17. 10.1186/s12859-016-1323-z. PMC5123296 27884101

[66] Nagam V. Early Detection of Temporal Lobe Epilepsy: Identification of Novel Candidate Genes and Potential Biomarkers Using Integrative Genomics Analysis. Open J Genet. 2020;10:65–81. 10.4236/ojgen.2020.104006.

[67] Szklarczyk D, Kirsch R, Koutrouli M, Nastou K, Mehryary F, Hachilif R, The STRING database in 2023: protein–protein association networks and functional enrichment analyses for any sequenced genome of interest. Nucleic Acids Res. 2023;51:D638–46. 10.1093/nar/gkac1000. 36370105 PMC9825434

[68] Huang Y, Xu W, Zhou R. NLRP3 inflammasome activation and cell death. Cellular and Molecular Immunology. 2021;18:2114–27. 10.1038/s41423-021-00740-6. 34321623 PMC8429580

[69] Shenoy AR, Wellington DA, Kumar P, Kassa H, Booth CJ, Cresswell P, GBP5 Promotes NLRP3 Inflammasome Assembly and Immunity in Mammals. Science (1979). 2012;336:481–5. 10.1126/science.1217141. 22461501

[70] Rathinam VAK, Vanaja SK, Waggoner L, Sokolovska A, Becker C, Stuart LM, TRIF licenses caspase-11-dependent NLRP3 inflammasome activation by gram-negative bacteria. Cell. 2012;150:606–19. 10.1016/j.cell.2012.07.007. 22819539 PMC3660860

[71] Yan J, Hedl M, Abraham C. An inflammatory bowel disease-risk variant in INAVA decreases pattern recognition receptor-induced outcomes. J Clin Invest. 2017;127:2192–205. 10.1172/JCI86282. 28436939 PMC5451247

[72] Paik S, Kim JK, Shin HJ, Park EJ, Kim IS, Jo EK. Updated insights into the molecular networks for NLRP3 inflammasome activation. Cell Mol Immunol. 2025;22:563–96. 10.1038/s41423-025-01284-9. 40307577 PMC12125403

[73] Wan P, Zhang S, Ruan Z, Liu X, Yang G, Jia Y, AP-1 signaling pathway promotes pro-IL-1β transcription to facilitate NLRP3 inflammasome activation upon influenza A virus infection. Virulence. 2022;13:502–13. 10.1080/21505594.2022.2040188. 35300578 PMC8942419

[74] Trstenjak-Prebanda M, Biasizzo M, Dolinar K, Pirkmajer S, Turk B, Brault V, Stefin B Inhibits NLRP3 Inflammasome Activation via AMPK/mTOR Signalling. Cells. 2023;12. 10.3390/cells12232731. PMC10798374 38067160

[75] Maher K, Kokelj BJ, Butinar M, Mikhaylov G, Manček-Keber M, Stoka V, A role for stefin B (cystatin B) in inflammation and endotoxemia. Journal of Biological Chemistry. 2014;289:31736–50. 10.1074/jbc.M114.609396. 25288807 PMC4231653

[76] Agrawal A, Balcı H, Hanspers K, Coort SL, Martens M, Slenter DN, WikiPathways 2024: next generation pathway database. Nucleic Acids Res. 2024;52:D679–89. 10.1093/nar/gkad960. 37941138 PMC10767877

[77] Tribolet L, Brice AM, Fulford TS, Layton DS, Godfrey DI, Bean AGD, Identification of a novel role for the immunomodulator ILRUN in the development of several T cell subsets in mice. Immunobiology. 2023;228:152380. 10.1016/j.imbio.2023.152380. 37031606

[78] Pham TAN, Clare S, Goulding D, Arasteh JM, Stares MD, Browne HP, Epithelial IL-22RA1-Mediated Fucosylation Promotes Intestinal Colonization Resistance to an Opportunistic Pathogen. Cell Host Microbe. 2014;16:504–16. 10.1016/j.chom.2014.08.017. 25263220 PMC4190086

[79] Chanchevalap S. Kruppel-like factor 5 is an important mediator for lipopolysaccharide-induced proinflammatory response in intestinal epithelial cells. Nucleic Acids Res. 2006;34:1216–23. 10.1093/nar/gkl014. 16500892 PMC1383625

[80] Seya T, Oshiumi H, Sasai M, Akazawa T, Matsumoto M. TICAM-1 and TICAM-2: toll-like receptor adapters that participate in induction of type 1 interferons. Int J Biochem Cell Biol. 2005;37:524–9. 10.1016/j.biocel.2004.07.018. 15618008

[81] Yano J, Noverr MC, Fidel PL. Cytokines in the host response to Candida vaginitis: Identifying a role for non-classical immune mediators, S100 alarmins. Cytokine. 2012;58:118–28. 10.1016/j.cyto.2011.11.021. 22182685 PMC3290723

[82] Viswanatha R, Ohouo PY, Smolka MB, Bretscher A. Local phosphocycling mediated by LOK/SLK restricts ezrin function to the apical aspect of epithelial cells. Journal of Cell Biology. 2012;199:969–84. 10.1083/jcb.201207047. 23209304 PMC3518218

[83] He J, Xie X, Xiao Z, Qian W, Zhang L, Hou X. Piezo1 in Digestive System Function and Dysfunction. Int J Mol Sci. 2023;24:12953. 10.3390/ijms241612953. 37629134 PMC10454946

[84] Farr Zuend C, Lamont A, Noel-Romas L, Knodel S, Birse K, Kratzer K, Increased genital mucosal cytokines in Canadian women associate with higher antigen-presenting cells, inflammatory metabolites, epithelial barrier disruption, and the depletion of L. crispatus. Microbiome. 2023;11:159. 10.1186/s40168-023-01594-y. 37491398 PMC10367425

[85] Ish-Shalom E, Meirow Y, Sade-Feldman M, Kanterman J, Wang L, Mizrahi O, Impaired SNX9 Expression in Immune Cells during Chronic Inflammation: Prognostic and Diagnostic Implications. The Journal of Immunology. 2016;196:156–67. 10.4049/jimmunol.1402877. 26608909

[86] Valido E, Capossela S, Glisic M, Hertig-Godeschalk A, Bertolo A, Stucki G, Gut microbiome and inflammation among athletes in wheelchair in a crossover randomized pilot trial of probiotic and prebiotic interventions. Sci Rep. 2024;14. 10.1038/s41598-024-63163-z. PMC11150429 38834634

[87] Wastyk HC, Fragiadakis GK, Perelman D, Dahan D, Merrill BD, Yu FB, Gut-microbiota-targeted diets modulate human immune status. Cell. 2021;184:4137–4153.e14. 10.1016/j.cell.2021.06.019. 34256014 PMC9020749

[88] Zheng J, Hoffman KL, Chen JS, Shivappa N, Sood A, Browman GJ, Dietary inflammatory potential in relation to the gut microbiome: Results from a cross-sectional study. British Journal of Nutrition. 2020;124:931–42. 10.1017/S0007114520001853. 32475373 PMC7554089

[89] Cauci S, Driussi S, Quadrifoglio F, Driussi S, Guaschino S, Isola M. Correlation of local interleukin-1beta levels with specific IgA response against Gardnerella vaginalis cytolysin in women with bacterial vaginosis. American Journal of Reproductive Immunology. 2002;47:257–64. 10.1034/j.1600-0897.2002.01096.x. 12148539

[90] Costa-Fujishima M, Yazdanpanah A, Horne S, Lamont A, Lopez P, Farr Zuend C, Nonoptimal bacteria species induce neutrophil-driven inflammation and barrier disruption in the female genital tract. Mucosal Immunol. 2023;16:341–56. 10.1016/j.mucimm.2023.04.001. 37121385 PMC13403981

[91] France M, Alizadeh M, Brown S, Ma B, Ravel J. Towards a deeper understanding of the vaginal microbiota. Nat Microbiol. 2022;7:367–78. 10.1038/s41564-022-01083-2. 35246662 PMC8910585

[92] Gentile GL, Rupert AS, Carrasco LI, Garcia EM, Kumar NG, Walsh SW, Identification of a cytopathogenic toxin from sneathia amnii. J Bacteriol. 2020;202. 10.1128/JB.00162-20. PMC7283592 32291280

[93] Garcia EM, Kraskauskiene V, Koblinski JE, Jefferson KK. Interaction of gardnerella vaginalis and vaginolysin with the apical versus basolateral face of a three-dimensional model of vaginal epithelium. Infect Immun. 2019;87. 10.1128/IAI.00646-18. PMC6434120 30692180

[94] Castro J, Martins AP, Rodrigues ME, Cerca N. Lactobacillus crispatus represses vaginolysin expression by BV associated Gardnerella vaginalis and reduces cell cytotoxicity. Anaerobe. 2018;50:60–3. 10.1016/j.anaerobe.2018.01.014. 29427630

[95] Castro J, França A, Bradwell KR, Serrano MG, Jefferson KK, Cerca N. Comparative transcriptomic analysis of Gardnerella vaginalis biofilms vs. planktonic cultures using RNA-seq. NPJ Biofilms Microbiomes. 2017;3:3. 10.1038/s41522-017-0012-7. 28649404 PMC5460279

[96] Decout A, Krasias I, Roberts L, Gimeno Molina B, Charenton C, Brown Romero D, Lactobacillus crispatus S-layer proteins modulate innate immune response and inflammation in the lower female reproductive tract. Nat Commun. 2024;15. 10.1038/s41467-024-55233-7. PMC11685708 39737998

[97] Swanson K V., Deng M, Ting JP-Y. The NLRP3 inflammasome: molecular activation and regulation to therapeutics. Nat Rev Immunol. 2019;19:477–89. 10.1038/s41577-019-0165-0. 31036962 PMC7807242

[98] Malet JK, Impens F, Carvalho F, Hamon MA, Cossart P, Ribet D. Rapid remodeling of the host epithelial cell proteome by the listeriolysin O (LLO) pore-forming toxin. Molecular and Cellular Proteomics. 2018;17:1627–36. 10.1074/mcp.RA118.000767. 29752379 PMC6072537

[99] Chakraborty S, Ganguli D, Nagaraja T, Gope A, Dey S, Pal A, Salmonella typhi serine threonine kinase T4519 induces lysosomal membrane permeabilization by manipulating toll-like receptor 2-Cystatin B-Cathepsin B-NF-κB-reactive oxygen species pathway and promotes survival within human macrophages. PLoS Pathog. 2025;21 4 April. 10.1371/journal.ppat.1013041. PMC11984733 40168426

[100] Greaney AJ, Leppla SH, Moayeri M. Bacterial exotoxins and the inflammasome. Front Immunol. 2015;6 NOV. 10.3389/fimmu.2015.00570. PMC4639612 26617605

[101] Roselletti E, Perito S, Gabrielli E, Mencacci A, Pericolini E, Sabbatini S, NLRP3 inflammasome is a key player in human vulvovaginal disease caused by Candida albicans. Sci Rep. 2017;7. 10.1038/s41598-017-17649-8. PMC5736597 29259175

[102] Riestra AM, Valderrama JA, Patras KA, Booth SD, Quek XY, Tsai CM, Trichomonas vaginalis induces NLRP3 inflammasome activation and pyroptotic cell death in human macrophages. J Innate Immun. 2018;11:86–98. 10.1159/000493585. 30391945 PMC6296884

[103] Peng Y, Xu Y, Li S, Shao M, Shen Z, Qi W. Mechanism of Vaginal Epithelial Cell Pyroptosis Induced by the NLRP3 Inflammasome in Vulvovaginal Candidiasis. American Journal of Reproductive Immunology. 2024;92. 10.1111/aji.13893. 38958245

[104] Abdul-Sater AA, Saïd-Sadier N, Padilla E V., Ojcius DM. Chlamydial infection of monocytes stimulates IL-1β secretion through activation of the NLRP3 inflammasome. Microbes Infect. 2010;12:652–61. 10.1016/j.micinf.2010.04.008. 20434582 PMC4074088

[105] Yang C, Lei L, Collins JWM, Briones M, Ma L, Sturdevant GL, Chlamydia evasion of neutrophil host defense results in NLRP3 dependent myeloid-mediated sterile inflammation through the purinergic P2X7 receptor. Nat Commun. 2021;12. 10.1038/s41467-021-25749-3. PMC8443728 34526512

[106] Duncan JA, Gao X, Huang MT-H, O’Connor BP, Thomas CE, Willingham SB, Neisseria gonorrhoeae Activates the Proteinase Cathepsin B to Mediate the Signaling Activities of the NLRP3 and ASC-Containing Inflammasome. The Journal of Immunology. 2009;182:6460–9. 10.4049/jimmunol.0802696. 19414800 PMC2722440

[107] Li LH, Lin JS, Chiu HW, Lin WY, Ju TC, Chen FH, Mechanistic Insight Into the Activation of the NLRP3 Inflammasome by Neisseria gonorrhoeae in Macrophages. Front Immunol. 2019;10:1815. 10.3389/fimmu.2019.01815. 31417575 PMC6685137

[108] Hua KF, Hsu HT, Huang MS, Chiu HW, Wong WT, Peng CH, Honokiol Exhibits Anti-NLRP3 Inflammasome and Antimicrobial Properties in Neisseria gonorrhoeaeInfected Macrophages. J Inflamm Res. 2024;17:3499–513. 10.2147/JIR.S454221. 38828053 PMC11144415

[109] Lin WY, Tsui JL, Chiu HW, Wong WT, Wu C, Hsu HT, Exploring Candesartan, an angiotensin II receptor antagonist, as a novel inhibitor of NLRP3 inflammasome: alleviating inflammation in Neisseria gonorrhoeae infection. BMC Infect Dis. 2024;24. 10.1186/s12879-024-10208-3. PMC11585111 39578786

[110] Yang M, Liu S, Cai J, Sun X, Li C, Tan M, Bile acids ameliorates lipopolysaccharide-induced endometritis in mice by inhibiting NLRP3 inflammasome activation. Life Sci. 2023;331. 10.1016/j.lfs.2023.122062. 37666389

[111] Chen S, Zhu L, Fang X, Appiah C, Ji Y, Chen Z, Alloferon Mitigates LPS-Induced Endometritis by Attenuating the NLRP3/CASP1/IL-1β/IL-18 Signaling Cascade. Inflammation. 2025;48:730–46. 10.1007/s10753-024-02083-6. 38913143

[112] Gomez-Lopez N, Romero R, Xu Y, Plazyo O, Unkel R, Leng Y, A Role for the Inflammasome in Spontaneous Preterm Labor with Acute Histologic Chorioamnionitis. Reproductive Sciences. 2017;24:1382–401. 10.1177/1933719116687656. 28122480 PMC5933090

[113] Gomez-Lopez N, Romero R, Galaz J, Xu Y, Panaitescu B, Slutsky R, Cellular immune responses in amniotic fluid of women with preterm labor and intra-amniotic infection or intra-amniotic inflammation. American Journal of Reproductive Immunology. 2019;82. 10.1111/aji.13171. PMC6788958 31323170

[114] Gomez-Lopez N, Romero R, Panaitescu B, Leng Y, Xu Y, Tarca AL, Inflammasome activation during spontaneous preterm labor with intra-amniotic infection or sterile intra-amniotic inflammation. American Journal of Reproductive Immunology. 2018;80. 10.1111/aji.13049. PMC6419509 30225853

[115] Faro J, Romero R, Schwenkel G, Garcia-Flores V, Arenas-Hernandez M, Leng Y, Intra-amniotic inflammation induces preterm birth by activating the NLRP3 inflammasome. Biol Reprod. 2019;100:1290–305. 10.1093/biolre/ioy261. 30590393 PMC6698670

[116] Yang D, Wang Z, Chen Y, Guo Q, Dong Y. Interactions between gut microbes and NLRP3 inflammasome in the gut-brain axis. Comput Struct Biotechnol J. 2023;21:2215–27. 10.1016/j.csbj.2023.03.017. 37035548 PMC10074411

[117] Gomez-Lopez N, Romero R, Xu Y, Garcia-Flores V, Leng Y, Panaitescu B, Inflammasome assembly in the chorioamniotic membranes during spontaneous labor at term. American Journal of Reproductive Immunology. 2017;77. 10.1111/aji.12648. PMC5429868 28233423

[118] Morrill S, Gilbert NM, Lewis AL. Gardnerella vaginalis as a Cause of Bacterial Vaginosis: Appraisal of the Evidence From in vivo Models. Front Cell Infect Microbiol. 2020;10. 10.3389/fcimb.2020.00168. PMC7193744 32391287

[119] Nicholls-Dempsey L, Badeghiesh A, Baghlaf H, Dahan MH. How does high socioeconomic status affect maternal and neonatal pregnancy outcomes? A population-based study among American women. Eur J Obstet Gynecol Reprod Biol X. 2023;20:100248. 10.1016/j.eurox.2023.100248. 37876770 PMC10590715

[120] Thermidor S, Gaballa D, Hentz R, Fishbein J, Vaidean G, Weinberg C, Clinical, Sociodemographic, and Neighborhood Characteristics Associated with Adverse Pregnancy Outcomes. J Womens Health. 2024;33:308–17. 10.1089/jwh.2023.0032. 38061042

[121] McHale P, Schlüter DK, Turner M, Care A, Barr B, Paranjothy S, How are socioeconomic inequalities in preterm birth explained by maternal smoking and maternal body mass index: A mediation analysis. Paediatr Perinat Epidemiol. 2024;38:142–51. 10.1111/ppe.13045. 38247280

[122] Jones EJ, Marsland AL, Kraynak TE, Votruba-Drzal E, Gianaros PJ. Subjective Social Status and Longitudinal Changes in Systemic Inflammation. Annals of Behavioral Medicine. 2023;57:951–64. 10.1093/abm/kaad044. 37549189 PMC10578390

[123] Harper DM, DeMars LR. HPV vaccines – A review of the first decade. Gynecol Oncol. 2017;146:196–204. 10.1016/j.ygyno.2017.04.004. 28442134

[124] Roy V, Jung W, Linde C, Coates E, Ledgerwood J, Costner P, Differences in HPV-specific antibody Fc-effector functions following Gardasil^®^ and Cervarix^®^ vaccination. NPJ Vaccines. 2023;8. 10.1038/s41541-023-00628-8. PMC10017795 36922512

[125] Di Pietro M, Filardo S, Grazia Porpora M, Recine N, Agnese Latino M, Sessa R. HPV/Chlamydia trachomatis co-infection: metagenomic analysis of cervical microbiota in asymptomatic women. New Microbiologica. 2018;41:1121–7138. 29313867

[126] Lebeau A, Bruyere D, Roncarati P, Peixoto P, Hervouet E, Cobraiville G, HPV infection alters vaginal microbiome through down-regulating host mucosal innate peptides used by Lactobacilli as amino acid sources. Nat Commun. 2022;13. 10.1038/s41467-022-28724-8. PMC8885657 35228537

[127] Gimeno-Molina B, Muller I, Kropf P, Sykes L. The Role of Neutrophils in Pregnancy, Term and Preterm Labour. Life. 2022;12. 10.3390/life12101512. PMC9605051 36294949

